# Docking-score landscapes shape active-learning performance across Vina, Glide, and SILCS

**DOI:** 10.1007/s10822-026-00927-x

**Published:** 2026-09-11

**Authors:** Joseph Chung, Aashish Bhatt, Jacob Ede Levine, Yi-Chun Lin, Mingtian Zhao, Alexander D. MacKerell Jr., Sunhwan Jo, Sai Chandra Kosaraju, Yun Lyna Luo

**Affiliations:** 1 Department of Biotechnology and Pharmaceutical Sciences, Western University of Health Sciences, Pomona, CA USA; 2 Department of Computer Science, California State Polytechnic University, Pomona, CA USA; 3 Department of Pharmaceutical Science, School of Pharmacy, University of Maryland, Baltimore, MD USA

**Keywords:** Active learning, Virtual screening, Chemprop, Vina, Glide, SILCS

## Abstract

The rapid expansion of large chemical libraries has created a need for virtual screening workflows that are both efficient and accurate. Active learning (AL) offers a scalable strategy by iteratively training surrogate models to prioritize promising compounds and reduce the number of required docking calculations. However, direct benchmarking of active-learning protocols across docking engines remains limited. In this study, we ask whether docking scores generated by different docking engines affect AL performance and what factors underlie these differences. To do so, we compare four active-learning virtual screening workflows, Vina-MolPAL, Glide-MolPAL, SILCS-MolPAL, and Schrödinger’s active-learning Glide, across multiple protein targets and library sizes. Performance was assessed by recovery of top-scoring molecules according to each workflow’s respective docking engine, score-prediction accuracy, chemical diversity, and computational cost. Vina-MolPAL achieved the highest top-1% recovery at a 1% batch size, whereas SILCS-MolPAL achieved comparable recovery at a larger batch size. Latent-embedding analyses suggest that the docking-score landscape strongly influences active-learning performance. In addition, combining active learning with SILCS provides a computationally efficient, membrane-aware approach for screening compounds at transmembrane binding sites.

**Supplementary Information:**

The online version contains supplementary material available at https://doi.org/10.1007/s10822-026-00927-x.

## Introduction

     Molecular docking has been widely used to identify potential drug candidates for proteins of interest [1, 2]. Docking programs evaluate multiple ligands within a binding site and prioritize them using scoring functions that approximate binding affinity. Over the past decade, virtual compound libraries have expanded rapidly [3–6], and now contain tens of billions of readily synthesizable molecules derived from available building blocks [5, 7–9]. Although virtual screening is far more cost-effective than wet-lab high-throughput screening, exhaustive docking of such large and continuously expanding chemical space is computationally prohibitive. To address this challenge, several groups have pioneered active-learning (AL) strategies that integrate machine learning with traditional docking-based workflows, enabling more efficient exploration of chemical libraries [10, 11].

Active learning has been widely applied in various scientific discovery problems [12–15]. In the context of virtual screening, a surrogate model is trained on the docking scores of a subset of compounds, and its predictions for the remaining library are used to exclude the least promising candidates from further evaluation. For instance, MolPAL, an active-learning workflow, iteratively trains on a small, docked subset of a chemical library using a directed message-passing neural network (D-MPNN) surrogate model, a variant of the MPNN model that operates on molecular graphs and outputs a prediction of molecular properties [10]. Another popular AL workflow, Schrödinger Inc.’s active-learning Glide (AL-Glide) DeepAutoQSAR model, uses both graph convolutional neural networks (GCNNs) and random forests to train iteratively on small batches of molecules [11]. In both approaches, the models predict the docking scores for the remaining compounds after each iteration and select the top-k scoring candidates for docking in the next round. This AL loop enables the recovery of high-ranking compounds by docking only a small fraction of the library.

Although MolPAL, AL-Glide, and other frameworks have been thoroughly benchmarked individually [10, 11, 16–18], to the best of our knowledge, there have been no direct comparisons across different frameworks using an identical dataset. It therefore remains unclear how docking scores generated by different docking engines affect AL performance and what factors underlie these differences.

In addition, evaluations of AL frameworks on membrane-embedded proteins, where ligands bind at the protein–membrane interface, remain rare. Over the past decade, advances in cryo-electron microscopy (cryo-EM) have led to a surge in structural data for transmembrane proteins with bound ligands [19, 20]. However, these lipid-exposed binding sites present unique challenges for both conventional and deep-learning-based docking programs, as most have been benchmarked primarily on soluble proteins [21–24]. SILCS (Site-Identification by Ligand Competitive Saturation) is a molecular dynamics (MD) simulation-based functional-group mapping approach [25]. Because its functional-group density maps, or FragMaps, are generated in a realistic membrane environment, SILCS-MC provides a more physically rigorous framework for compound screening at protein–membrane interfaces [26–28]. However, SILCS-MC docking currently requires approximately 60 seconds per compound on a single GPU. We therefore evaluated whether active learning could be used to reduce the computational cost of SILCS-MC screening and compared its performance with Vina- and Glide-based AL workflows.

## Results and discussion

### Active learning workflow validation

The MolPAL workflow uses the directed message-passing neural network (D-MPNN) implemented in Chemprop 1.1.0 for model training and prediction [29, 30]. In the main text, we present results obtained using the original MolPAL implementation with Chemprop 1.1.0. Because Chemprop v2 became available during the preparation of this manuscript [31], we also report results obtained using Chemprop v2 in the Supplementary Information.

Because we customized the MolPAL workflow for our local GPU cluster by integrating various docking engines, Chemprop training, and acquisition through in-house scripts, we first validated our implementation by reproducing the published MolPAL results on the Enamine50k dataset [7, 10]. We then evaluated MolPAL on 50,000 molecules randomly selected from the ChemBridge CORE Library Stock to determine whether comparable performance could be achieved on a different chemical library. For this benchmark, we used a D-MPNN surrogate model with greedy acquisition, in which the highest-ranked molecules were selected as the next docking batch at each iteration, because this strategy performed well in previous MolPAL benchmarks [10]. Following the original MolPAL protocol, the model was retrained from scratch at the beginning of each iteration using all accumulated training data.

As shown in Figure S1, our in-house workflow recovered 70.0 ± 1.79% of the top 500 molecules in the Enamine50k dataset after evaluating 3000 molecules, corresponding to approximately 6% of the library. This result is consistent with the previously reported recovery rate of 71.5 ± 1.8% [10]. Using the same protocol, we achieved a higher recovery rate of 87.93 ± 0.57% on the ChemBridge 50k dataset. Together, these results support the validity of our customized implementation and benchmark setup.

**Fig. 1:**
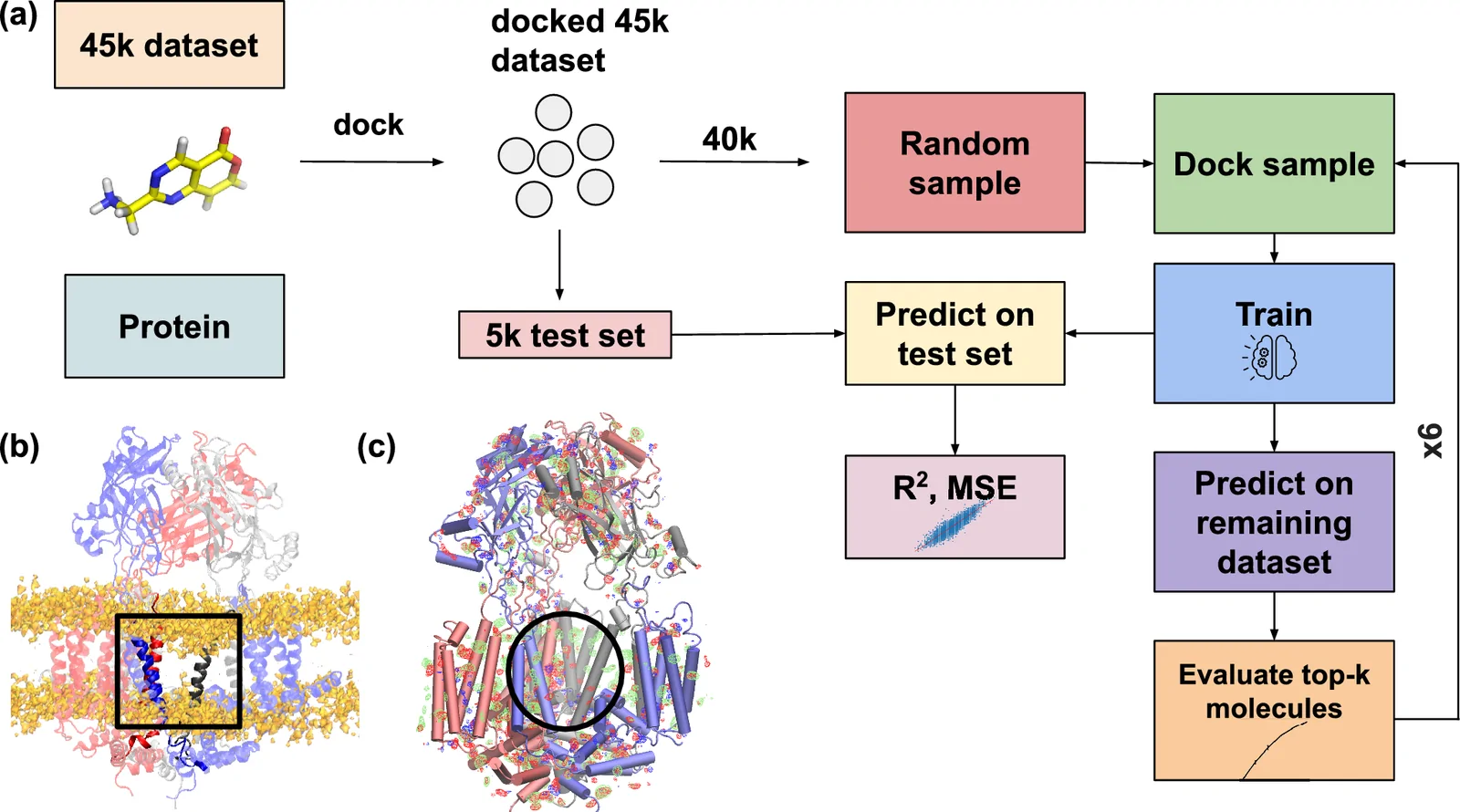
Active learning workflow and PIEZO2 pore model. **a** Overview of the benchmark workflow. **b** PIEZO2 open pore model used for Vina and Glide docking. Protein backbone is shown in newcartoon representation and colored by subunit. POPC lipid headgroups are shown in yellow surface model. The cubic docking box of 18 x 18 x 18 Å$^{3}$ is used for both Vina and Glide docking. Lipids were included during docking. **c** SILCS FragMaps of PIEZO2 open pore model. The protein backbone is shown in cartoon representation and colored by subunit. FragMaps are shown as colored meshes. For clarity, only three types are displayed: the generic apolar map (green), the generic hydrogen-bond donor map (blue), and the generic hydrogen-bond acceptor map (red). The SILCS-MC docking site is indicated by a black circle of radius of 18 Å

### Dataset

We generated three docking-score datasets to serve as ground-truth data for evaluating active-learning performance. Here, “ground truth” refers to the scores produced by the respective docking engines rather than experimentally measured binding affinities. The first binding site is located within the open pore of the PIEZO2 ion channel (Fig. 1b). The open-pore conformation was obtained from our previous molecular dynamics simulations [32]. We retained the surrounding POPC lipids as part of the receptor because the PIEZO2 open pore is formed by three inner-pore helices with intercalated lipids between them. The Vina dataset was generated using AutoDock Vina [33], the Glide dataset using Schrödinger’s Glide SP [34], and the SILCS dataset using SILCS-MC docking [35] with our precomputed PIEZO2 FragMaps (Fig. 1c).

We then extended our comparisons by generating Vina and Glide docking-score datasets for two additional targets: the kinase ALK2 and the voltage-gated ion channel HCN1 to evaluate the generalizability of our findings across distinct protein families. For ALK2, the binding site was defined as the solvent-exposed ATP-binding site within the kinase domain [36]. For HCN1, the binding site was defined at the interface between the lipid bilayer and the cytoplasmic side, representing the same pocket occupied by ivabradine in the HCN1 structure [37].

We first report benchmarking results using three independent replicas of a 45,000-compound dataset, each consisting of a 40k active-learning pool and a 5k held-out test set (Fig. 1a). Each replica was randomly selected from the Chembridge library. To assess whether library size influences the benchmarking outcomes, we also evaluated Vina and Glide using the full in-stock Chembridge library, consisting of 1.3 million compounds. For ALK2 and HCN1, active learning performance was evaluated for Vina and Glide using a 40k and 20k compound datasets, respectively, sampled from the 1.3-million-compound ChemBridge library. In addition, the performance of the three MolPAL-based workflows was compared with Schrödinger’s AL platform developed by Yang et al. [11]. The general AL workflow is illustrated in Fig. 1. Greedy acquisition was used throughout this benchmark study. However, for Glide-MolPAL, Upper Confidence Bound (UCB) acquisition was also tested to rule out the acquisition strategy as the cause of its lower performance.

### Top-*k*% molecule recovery rate with 1% and 5% batch sizes

**Fig. 2:**
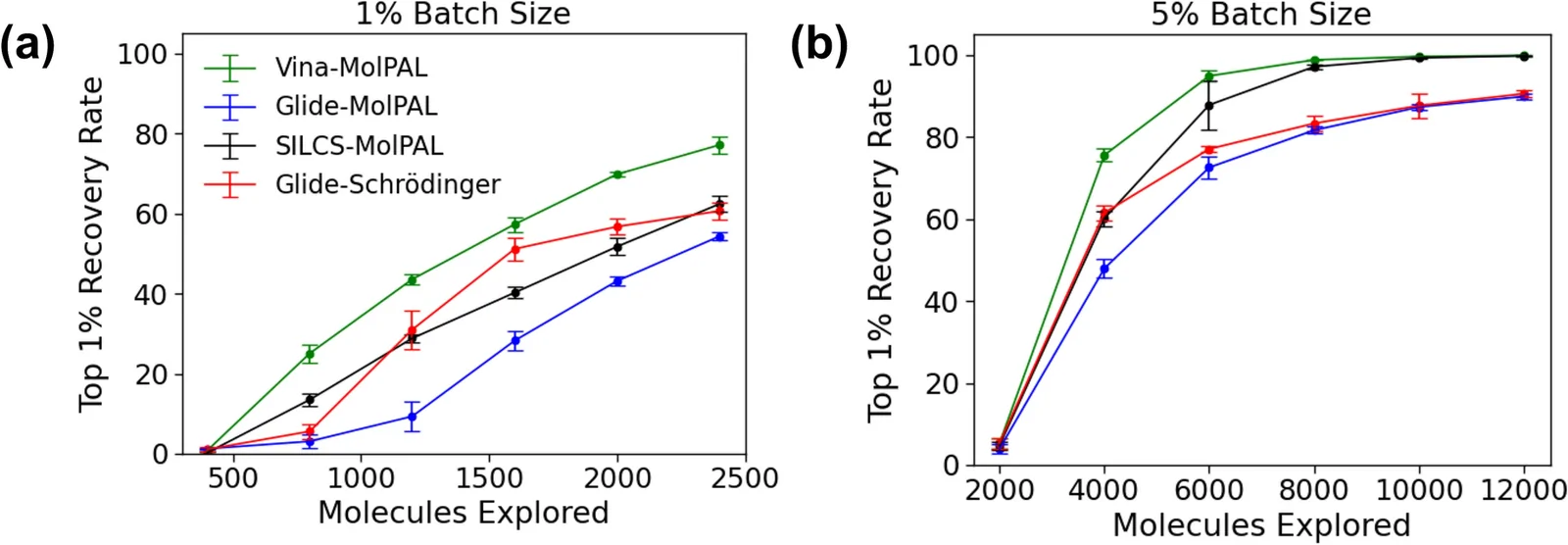
Percentage of top 1% scoring SMILES recovered as a function of the number of docked molecules. **a** Results from six iterations of 1% batch size (400 molecules per batch).** b** Results from six iterations of 5% batch size (2000 molecules per batch). Error bars are standard deviation from three replicas using random initial batches. (See Figure S2 for the corresponding results obtained from Chemprop v2)

We use the percent recovery rate of the top-scoring 1% of molecules as a performance metric because it is directly relevant to real-world virtual screening campaigns, where identifying the highest-ranking compounds is the primary objective. Using a 1% batch size of 40,000-compound library and six training iterations, corresponding to docking 6% of the entire library, MolPAL trained on the Vina dataset (green line) outperformed the other three workflows, achieving the highest recovery rate of 77.17 ± 2.08 *%* for the top 1% scoring SMILES strings after six iterations. MolPAL trained on the SILCS-MC dataset and AL-Glide followed, with recovery rates of 62.42 ± 2.06 *%* and 60.67 ± 2.08%, respectively (black and red lines). However, MolPAL trained on the Glide dataset yielded the lowest recovery rate of 54.33 ± 0.94 *%* (blue line) (Fig. 2a).

The optimal batch size depends on the size of the virtual screening library and the available computational resources. A 1*%* batch size for the 40,000-compound library corresponds to adding only 400 docking scores to the training set at each iteration. We hence also evaluated model performance using a 5*%* batch size, corresponding to docking 2000 molecules per iteration. As expected, all models exhibited overall performance improvements with the larger batch size (Fig. 2b). Vina-MolPAL and SILCS-MolPAL achieved the highest recovery rates, with 99.92 ± 0.12% and 99.83 ± 0.12%, respectively. Performance plateaued after the fourth iteration, when 20*%* of the library had been docked. In contrast, the AL models trained with the Glide dataset converged more slowly and yielded lower recovery rates of 89.92 ± 0.66% (MolPAL) and 90.58 ± 0.92% (Schrödinger’s active-learning Glide). Among the four datasets, SILCS-MolPAL showed the largest improvement when increasing batch sizes. All models were additionally evaluated for their recovery rate across varying top-*k*% scoring SMILES thresholds for both a 1% and 5% batch size. The trends observed in Fig. 2 remained consistent across different top-*k*% cutoffs (Figs. S3, S4).

To evaluate whether the relatively poor performance of Glide-MolPAL was driven by the acquisition metric, we repeated the AL protocol for both the 1% and 5% batch sizes using the Upper Confidence Bound (UCB) acquisition function, which has previously been reported by *Graff et al.* [10] to outperform greedy acquisition in some settings. As shown in Table S1, recovery rates were largely insensitive to the choice of acquisition function, indicating that the acquisition metric is unlikely to be the primary cause of Glide-MolPAL’s lower performance.

Across the three docking programs (Vina, Glide, SILCS-MC), MolPAL performed best when trained on Vina docking scores, followed by SILCS-MC scores. Schrödinger’s AL-Glide exhibited performance comparable to Glide-MolPAL, indicating that the type of docking scores, rather than the AL algorithm itself, is the primary factor driving performance differences in our study. These results demonstrate that the choice of docking method plays a critical role in overall AL virtual screening performance.

### Model convergence on hold-out test set

**Fig. 3:**
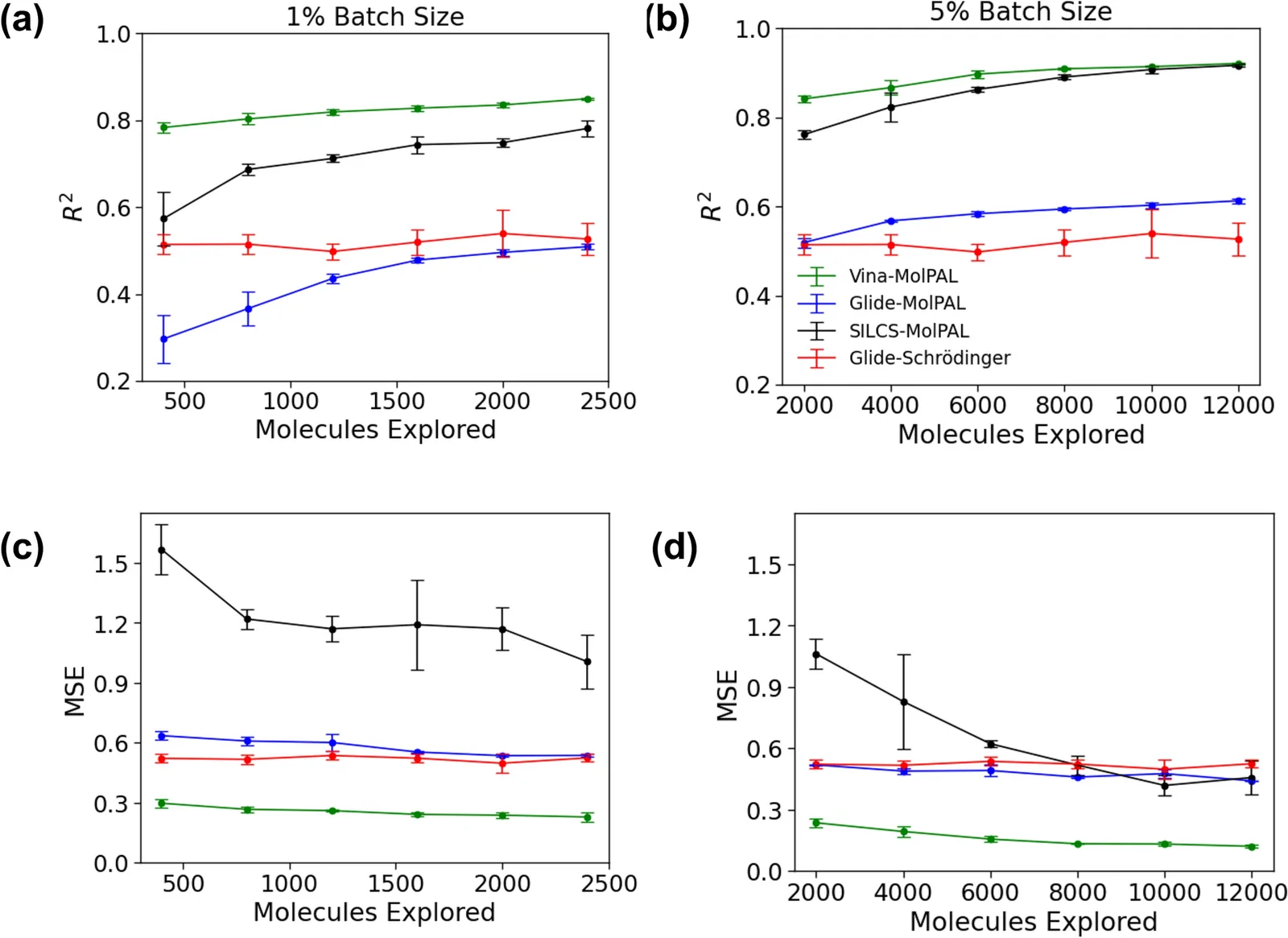
Model convergence on test set $R^2$ and MSE for MolPAL using Vina, Glide, and SILCS datasets and AL-Glide as a function of the number of screened molecules over three replicas. **a**,** c** $R^2$ and MSE for a 1% batch size, respectively** b**,** d** $R^2$ and MSE for a 5% batch size, respectively

To better understand the difference in model performance, we analyzed the convergence of $R^2$ and mean squared error (MSE) between predicted scores and docking scores over iterations using a hold-out test set of 5000 unseen SMILES strings (Fig. 3). All MolPAL-based AL workflows exhibit progressively increasing $R^2$ and decreasing MSE as more molecules are explored, indicating that AL consistently improves model generalization as additional labeled data are incorporated. In contrast, AL-Glide did not show measurable improvements in either $R^2$ or MSE with the addition of new labeled data.

With a 1% batch size, MolPAL trained on Vina (green), SILCS-MC (black), and Glide (blue) datasets achieved final $R^2$ values of 0.85 ± 0.00, 0.78 ± 0.02, 0.51 ± 0.01, respectively, while AL-Glide (red) achieved a final $R^2$ of 0.53 ± 0.04 (Fig. 3a). A similar trend was observed with the 5% batch size, with overall improvements across all models: Vina-MolPAL, SILCS-MolPAL, Glide-MolPAL, and AL-Glide achieved final $R^2$ values of 0.92 ± 0.00, 0.92 ± 0.00, and 0.61 ± 0.01, 0.53 ± 0.04, respectively (Fig. 3b).

**Fig. 4:**
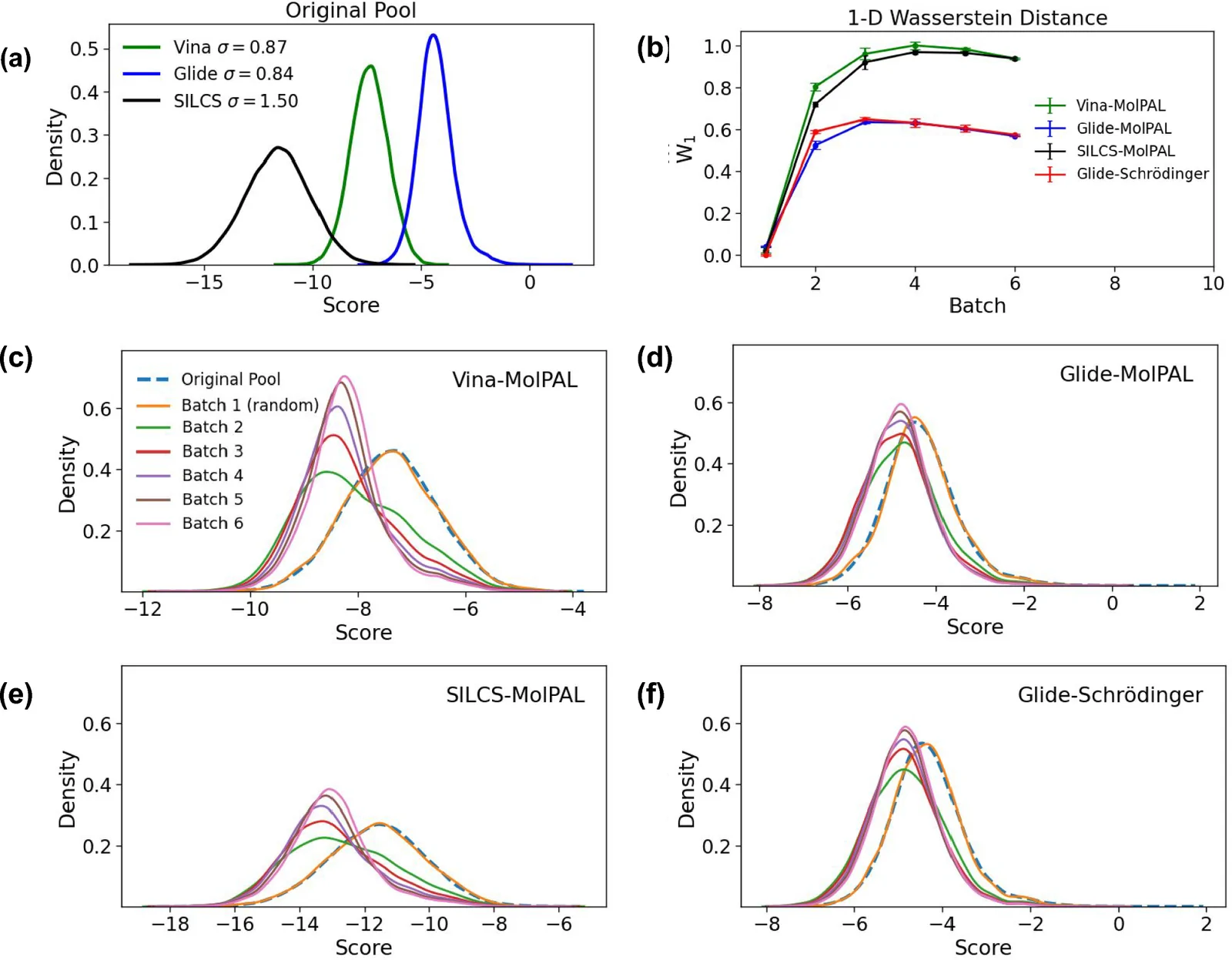
Docking score distributions. **a** Docking score distributions of the 40,000 SMILES dataset (i.e., the original pool) from Vina, Glide, and SILCS. **b** 1D Wasserstein distance (*W*$_1$) was calculated from the original pool to the distribution of each of the accumulated batches across three replicas using a 5% batch size. **c–f** Dotted lines represent the distributions of the original pool, while bold lines indicate the distributions of the accumulated training sets across batches from replica 1 using a 5% batch size. The panels correspond to Vina-MolPAL, Glide-MolPAL, SILCS-MolPAL, and AL-Glide, respectively (for 1% batch, see Figure S5)

Figure 3c shows the corresponding MSE on the same test set for the 1% batch size. Consistent with the $R^2$ trends, Vina-MolPAL produces the lowest MSE throughout the iterations. Interestingly, SILCS-MolPAL (black line) maintains a relatively high MSE (1.01 ± 0.13) despite achieving a high $R^2$. A similar trend is observed for the 5% batch size exploration (Fig. 3d). This apparent discrepancy can be explained by the larger variance in the SILCS-MC docking score (Fig. 4a). Because $R^2 = 1 - \frac{MSE}{\sigma ^2}$, a larger variance ($\sigma ^2$) can yield a higher $R^2$ for the same MSE. Notably, SILCS-MolPAL with the 5% batch size exhibits a substantial reduction in MSE up to the fifth iteration, ultimately converging to a final MSE comparable to that of Glide-MolPAL and AL-Glide. This result further indicates that SILCS-MolPAL benefits more than the other models from an increased batch size.

### Score distribution over iterations

To better understand differences in SMILES recovery rate and score prediction accuracy across four AL workflows, we monitored the score distributions after each training iteration (Fig. 4c–f). All models progressively enrich for higher-scoring molecules over successive active-learning batches, as reflected by systematic leftward shifts in the score distributions (i.e., more negative scores indicate better binding). In all tested workflows, the score distributions shift substantially toward more favorable values from the initial random batch (batch 1, orange) to batch 2 (green). From batches 3–6, the distributions become narrower and increasingly skewed toward strong binders, indicating incremental prioritization of high-affinity candidates.

To quantitatively assess sequential enrichment of high-scoring molecules across all models, we applied the Z-score normalization to our datasets, and computed the 1D Wasserstein distance (*W*$_1$) between the original pool distribution and the score distribution of each accumulated batch (Fig. 4b). A larger *W*$_1$ value indicates a greater distributional shift from the original pool, and thus stronger enrichment of high-affinity compounds. As shown in Fig. 4b, SILCS-MolPAL and Vina-MolPAL exhibit the strongest enrichment, surpassing the rest of the models by a substantial margin. This is followed by Glide-Schrödinger and Glide-MolPAL, exhibiting nearly identical shifts.

### Latent embedding and gradient analysis

**Fig. 5:**
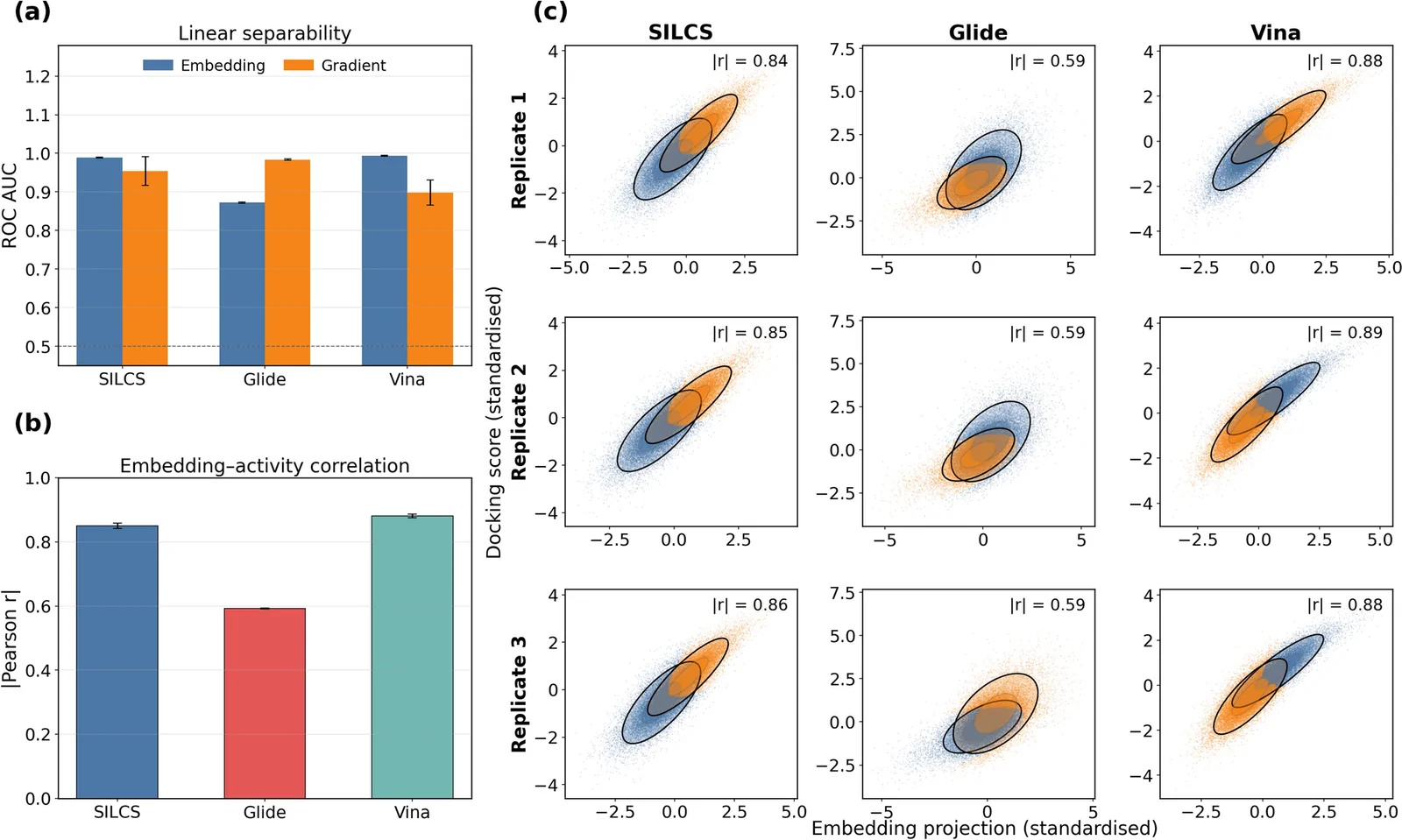
Molecule embedding analysis learned from Chemprop.** a** Linear-SVM classification of top versus bottom quartile binders (by ground-truth docking score) using the MolPAL’s molecule embedding and the gradient of the last layer from the last iteration of active learning using a 1% batch size. Bars show mean five-fold ROC-AUC over three replicates (± SD), dashed line marks chance.** b** Absolute Pearson correlation between docking score and a score-aligned 1-D projection of the embedding (mean ± SD).** c** Unsupervised two-component Gaussian mixture in the (embedding projection, docking score) plane; points colored by component, ellipses give the 1*σ * and 2*σ * covariances, inset reports |*r*|

To further understand how docking-score landscapes generated by different docking engines affect active-learning performance, we examined what the Chemprop surrogate model learns from each docking-score dataset. In the MolPAL workflow, Chemprop serves as a machine-learning surrogate for the docking engine: it takes each ligand SMILES as a molecular graph, converts it into a learned molecular representation, and predicts the docking score that would otherwise be obtained from Vina, Glide, or SILCS-MC. Therefore, if the docking scores from a given engine follow chemically learnable trends, Chemprop should be able to organize molecules in its latent space according to their predicted docking scores.

To test this, we extracted the final-layer molecular embeddings and the corresponding loss gradients from the last active-learning iteration using a 1% batch size, and evaluated how well each representation retained docking-score information (Fig. 5). The molecular embedding reflects the chemical features that Chemprop has learned to use for score prediction, whereas the loss gradient reflects the immediate training signal that drives model optimization.

The rationale is that, if Chemprop has learned a stable molecular representation organized by docking score, then high- and low-scoring molecules should be separable from the embedding using a simple linear classifier, and the docking-score trend should be recoverable from low-dimensional projections of the embedding. In contrast, if docking-score information is present in the loss gradients but weakly encoded in the molecular embedding, this suggests that Chemprop receives a score-dependent training signal but does not effectively consolidate that signal into a stable, score-organized latent representation.

Using a linear-kernel SVM trained to distinguish low-scoring from high-scoring molecules based on their docking scores (see Methods), the SILCS and Vina embeddings achieved mean AUCs of *0.989 ± 0.002* and *0.994 ± 0.001*, respectively (Fig. 5a). These results indicate that a simple linear boundary can almost perfectly separate the two score classes within the Chemprop embedding space. In contrast, the Glide embedding achieved a lower AUC of *0.872 ± 0.002*, suggesting substantially greater intermixing of low- and high-scoring molecules in the learned representation.

Interestingly, the gradient-based representations showed the opposite trend. Glide achieved the highest gradient AUC of *0.983 ± 0.002*, whereas Vina and SILCS achieved lower values of *0.899 ± 0.033* and *0.954 ± 0.037*, respectively. This contrast suggests that the Glide-trained Chemprop model receives a docking-score-dependent learning signal during optimization, as reflected in the gradient, but that this signal is less effectively organized into the final molecular embedding.

This weaker score organization in the Glide embedding was also observed in two complementary analyses. First, we projected each embedding onto the one-dimensional direction most aligned with the docking score and computed the absolute Pearson correlation between this projection and the reference docking score (Fig. 5b). The SILCS and Vina embeddings showed strong correlations of *0.850 ± 0.008* and *0.881 ± 0.006*, respectively, whereas the Glide embedding showed a weaker correlation of *0.593 ± 0.001*. Thus, even the most score-aligned direction in the Glide embedding tracks the docking-score trend less effectively than the corresponding directions in the SILCS and Vina embeddings.

Second, we fitted an unsupervised two-component Gaussian mixture model in the plane defined by the score-aligned embedding projection and the docking score (Fig. 5c). For SILCS and Vina, the two components separated into relatively distinct low- and high-scoring regions. In contrast, the Glide components showed greater overlap, indicating that the Glide embedding does not provide a similarly clean separation between low- and high-scoring molecules. Consistent with this observation, UMAP visualizations of the Chemprop embeddings during active learning showed that molecules selected by the Vina- and SILCS-trained models became concentrated in specific regions of the embedding space, whereas molecules selected by the Glide-trained model remained more broadly dispersed (Figs. S6, S7, S8).

Taken together, these analyses indicate that the Glide-trained Chemprop model encodes docking-score information less cleanly in its final molecular embedding than the Vina- and SILCS-trained models. We interpret this behavior as a possible consequence of a less chemically learnable or more heterogeneous Glide docking-score landscape. Although docking-score information is present in the Glide gradient, it appears to be less effectively incorporated into a stable, score-organized embedding. Because MolPAL acquisition relies on Chemprop predictions derived from this learned representation, weaker separation between low- and high-scoring molecules may contribute to the lower recovery rate and reduced score-prediction accuracy observed for Glide-MolPAL.

It is important to emphasize that “less learnable” in this context does not imply lower accuracy in predicting experimental binding affinity. Rather, it means that Glide docking scores are less predictable from SMILES-derived two-dimensional molecular graph representations in the Chemprop surrogate model. This suggests that Glide scores may depend more strongly on factors that are not explicitly encoded in the molecular graph, such as docked pose geometry or protein–ligand contacts. Therefore, the significance of our study is not to rank docking programs by their physical accuracy, but to evaluate the compatibility between machine-learning surrogate models and docking engines in active-learning virtual screening. In this context, more training data may be required for Chemprop to learn Glide scores than Vina or SILCS-MC scores.

### **Chemical diversity over iterations**

In addition to achieving a high top-*k*% molecule recovery rate, chemical diversity among docked molecules is desirable, as it enables broader exploration of chemical scaffolds during a virtual screening campaign. In our benchmarks, greedy acquisition was applied to all models, as it has been shown to outperform other acquisition strategies across multiple test sets [10]. However, greedy acquisition selects compounds solely based on best predicted docking scores at each iteration, without considering chemical diversity.

**Fig. 6:**
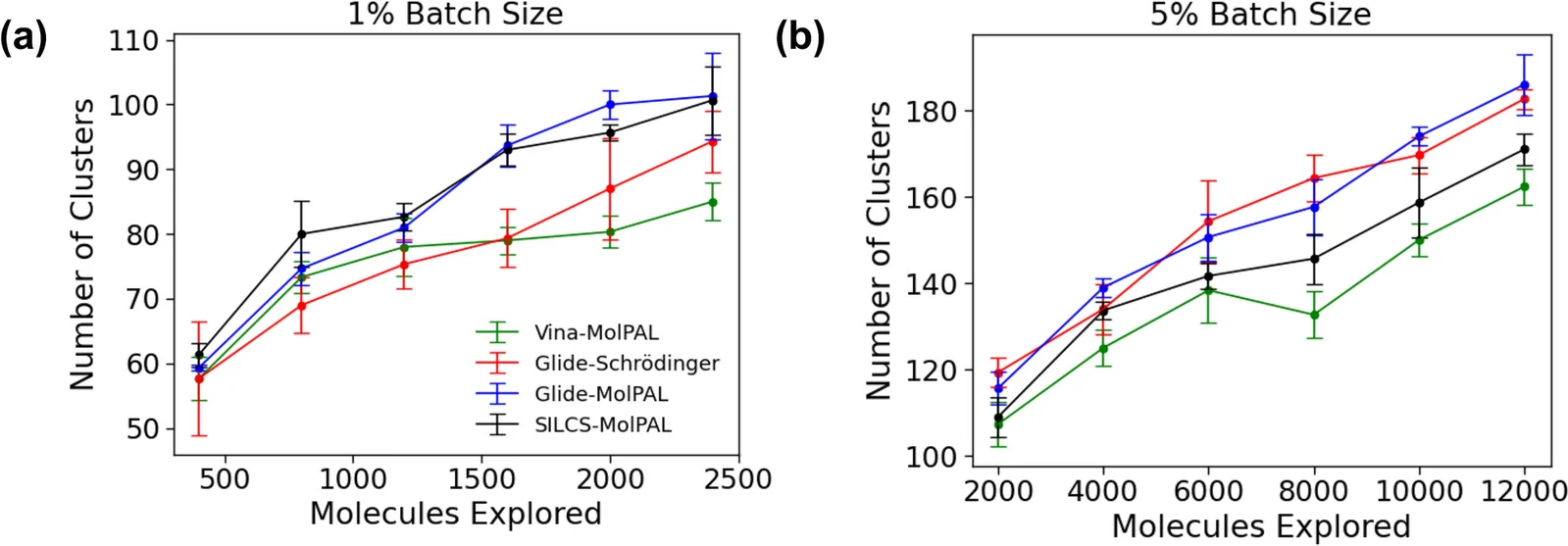
Diversity of molecules in accumulated batches as function of molecules explored. Molecules in accumulated batches were clustered using Butina Clustering using their Morgan fingerprints with a distance cutoff of 0.8.** a** 1% Batch Size (400 molecules)** b** Batch Size (2k molecules)

To compare diversity across the four models, we quantified the number of clusters of similar molecules in the accumulated batches. Regardless of batch size, the Vina–MolPAL workflow exhibited the lowest number of clusters, whereas MolPAL trained on the Glide dataset consistently produced the highest number of clusters (Fig. 6). Notably, the ranking of diversity across the four models is inversely correlated with recovery rate: the higher predictive performance in score prediction is associated with lower chemical diversity. This trend reflects the well-known exploration–exploitation trade-off in active learning. Because greedy acquisition more aggressively exploits high-affinity regions of chemical space in the MolPAL–Vina workflow, exploration of structurally diverse or high-uncertainty compounds becomes limited.

### Effect of library size

Lastly, to assess whether the results obtained from the 40k dataset are applicable to larger libraries, we docked the entire 1.3 million-compound ChemBridge in-stock library using Vina and Glide, with 10,000 compounds randomly held out as an external test set. SILCS-MolPAL was not included in this benchmark because brute-force docking of the full library with SILCS-MC is computationally prohibitive, further underscoring the need for active learning to reduce docking cost. Benchmarking on the remaining 1.29 million compounds was therefore limited to three active-learning frameworks.

**Fig. 7:**
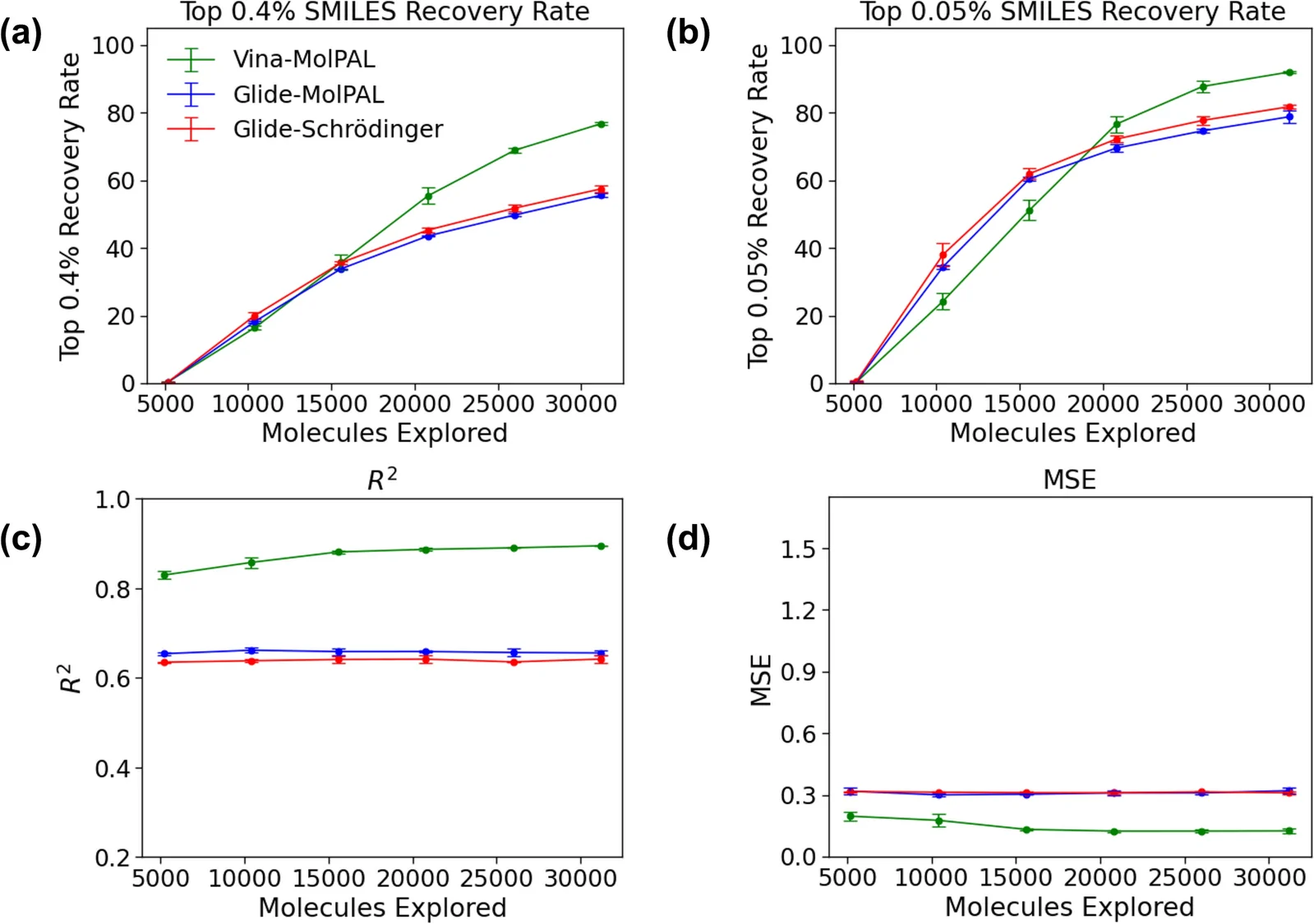
Performance metrics on a 1.29 M dataset. Performance metrics were evaluated using a 1.29M dataset and a 0.4% batch size (5200 molecules) for Vina-MolPAL, Glide-MolPAL, and AL-Glide.** a** Percentage of top 0.4% scoring SMILES strings as a function of number of docked molecules over three replicas. **b** Percentage of top−0.05% scoring SMILES strings as a function of number of docked molecules over three replicas.** c**$R^2$ as a function of number of screened molecules over three replicas. **d** MSE as a function of number of screened molecules over three replicas

For this large-scale benchmark, a 0.4% batch size (5200 molecules) was used, which is consistent with the original MolPAL study for libraries of similar scale [10]. After six docking-training iterations (2.5% of the library evaluated), Vina-MolPAL consistently achieved the highest recovery rate across all top-*k*% thresholds (0.05–1%), with highest $R^2$ and the lowest MSE relative to the Glide-based workflows. Glide-MolPAL and AL-Glide showed comparable performance across all metrics (Figs. 7, S9, and S10). Importantly, the relative ranking of workflows observed in the smaller 40k-compound benchmark is preserved in the 1.29 million–compound library. Trends in chemical diversity and score distribution shifts (Fig. S11) also remain consistent.

### Effect of protein targets

Finally, we investigated whether the recovery trends observed for PIEZO2 were reproducible across other biological targets. Additional active-learning benchmarks were performed for the ATP-binding site of the ALK2 kinase and a membrane-embedded binding site in the HCN1 ion channel using randomly sampled datasets of 40,000 and 20,000 compounds, respectively, drawn from the 1.3-million-compound ChemBridge library. For both targets, Vina-MolPAL consistently achieved higher recovery rates than Glide-MolPAL (Fig. S12), indicating that the lower learnability of the Glide docking-score landscape observed for PIEZO2 was also reproduced for ALK2 and HCN1. Together, these results suggest that the docking-score source is a major determinant of AL performance across the targets examined.

### Computational cost comparison

**Fig. 8:**
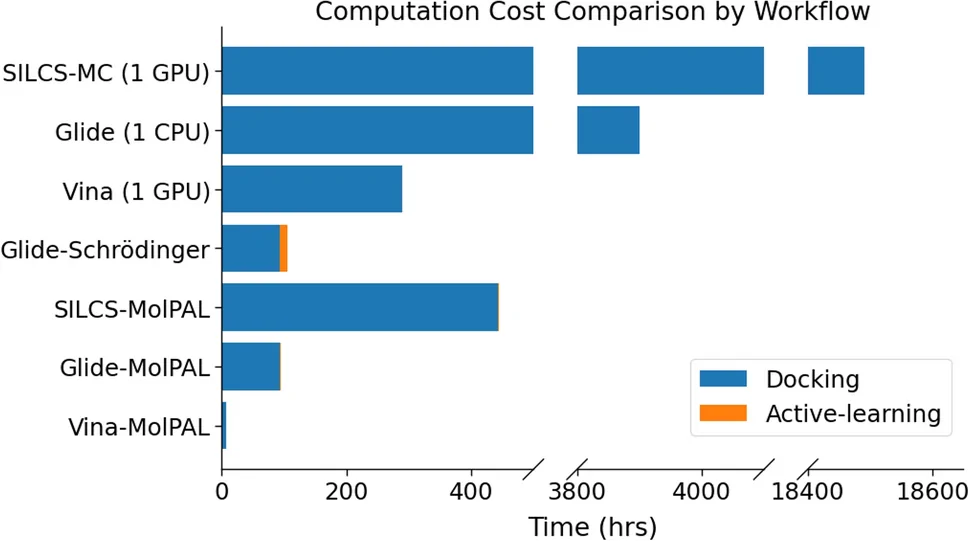
Computational cost comparison of docking and AL workflows. Total docking time for 1,300,000 molecules is shown for Vina, Glide, and SILCS, while total time for six AL iterations (0.4% batch size) is shown for Glide-Schrödinger, SILCS-MolPAL, Glide-MolPAL, and Vina-MolPAL. Vina and SILCS docking were run on a single GPU, Glide docking on a single CPU, MolPAL on a single CPU and GPU, and Schrödinger AL on a single CPU. Glide docking and Schrödinger AL runs were performed on Intel Xeon w9-3495X CPU (4.8 GHz), and all other computations were executed on an NVIDIA RTX A4500 GPU and an Intel Xeon Silver 4310T CPU (2.30 GHz)

Figure 8 and Table 1 summarize the computational cost associated with each combination of docking method and AL model. Without AL, the GPU version of SILCS-MC is 60-fold slower than the GPU version of Vina. Although SILCS-MC docking scores have demonstrated accuracy comparable to alchemical free energy methods in certain systems [23, 38], the high computational cost currently limits its practical application primarily to the lead-optimization stage. Glide was executed on a single CPU in this study and is therefore substantially slower than the GPU-accelerated Vina implementation.

When comparing the AL frameworks under their default settings, MolPAL running on an NVIDIA A4500 GPU card completes training on 5000 molecules over six iterations in less than four hours, substantially faster than Schrödinger’s DeepAutoQSAR model training. Considering both the computational cost and the performance in recovering top-ranked molecules, combination of Vina docking and MolPAL provides the optimal choice for AL virtual screening (Fig. 8).

However, the recovery rate reflects only the efficiency of the AL process and does not account for the intrinsic reliability of the underlying docking scores. In this study, we demonstrated that when combined with MolPAL, the SILCS-MC AL workflow achieved a 99.83% recovery rate for the top-1% scoring compounds by docking only 6*%* of a 40,000-compound library, corresponding to 18-fold speed-up (Table 1). SILCS-MolPAL framework is particularly advantageous for challenging targets, such as the transmembrane binding site of PIEZO2 ion channel used in this study, where SILCS-MC provides more realistic modeling of heterogeneous membrane environments. By integrating SILCS-MC with MolPAL, the more computationally expensive docking method becomes feasible for large-scale virtual screening on a local GPU cluster.

## Conclusions and perspectives

In this study, we performed a systematic comparison of active-learning (AL) virtual screening workflows by integrating three docking engines, AutoDock Vina, Schrödinger Glide, and SILCS-MC, with two machine-learning frameworks, MolPAL (Chemprop v1 and v2) and Schrödinger’s DeepAutoQSAR. Using identical chemical libraries at two dataset scales, 40k and 1.30M compounds, together with consistent evaluation metrics, we benchmarked workflow performance at a transmembrane binding site in terms of top-scoring molecule recovery, docking-score prediction accuracy, chemical diversity, and computational efficiency. A concise summary of the performance of these workflows is provided in Table 1.

**Table 1:** Results summary of Vina, Glide, and SILCS-based workflows

Workflow	*R*^2^ (1%)	Top-1% recovery (1%)	*R*^2^ (5%)	Top-1% recovery (5%)	Top−0.4% recovery (1.3 M)	*R*^2^ (1.3 M)	Speed-up (5%)	Speed-up (0.4%)
Vina-MolPAL	0.85 ± 0.00	77.17 ± 2.08%	0.92 ± 0.00	99.92 ±0.12%	77.15 ±0.35%	0.89 ±0.00	×11	×40
Glide-MolPAL	0.51 ± 0.01	54.33 ± 0.94%	0.61 ± 0.01	89.92 ±0.66%	56.12 ±0.54%	0.66 ± 0.01	×18	×42
SILCS-MolPAL	0.78 ± 0.02	62.42 ± 2.06%	0.92 ± 0.00	99.83 ±0.12%	N/A	N/A	×18	×42
Glide-Schrödinger	0.53 ± 0.04	60.67 ± 2.08%	0.53 ± 0.04	90.58 ± 0.92%	58.00 ±1.00%	0.64 ± 0.01	×12	×37

Our results show that Vina-MolPAL achieved the highest recovery rate across different top-*k*% thresholds and dataset sizes. Latent embedding analysis suggests that, in this study Glide docking scores are less readily predicted from SMILES-derived two-dimensional molecular graph representations by the Chemprop surrogate model. This interpretation is further supported by the consistent higher recovery rates achieved by Vina-MolPAL relative to Glide-MolPAL when applied to two distinct targets, ALK2 and HCN1. Importantly, this observation does not imply lower physical accuracy of Glide in predicting experimental binding affinity. Rather, it indicates that Glide scores may depend more strongly on factors not explicitly encoded in the molecular graph. Therefore, more training data or more expressive surrogate models may be required to learn Glide score landscapes as effectively as Vina or SILCS-MC score landscapes.

Encouragingly, SILCS-MolPAL achieved recovery rates comparable to those of Vina-MolPAL at a 5% batch size, suggesting a practical strategy for efficiently screening compounds at transmembrane binding sites. Although AL-based virtual screening was initially developed to enable the exploration of ultra-large chemical libraries containing billions of compounds, the synthesis and acquisition of make-on-demand compounds remain costly and time-consuming. In practice, large in-stock libraries containing millions of compounds may therefore be more accessible for exploratory studies. At this scale, the accuracy and physical realism of the docking method become increasingly important relative to computational speed [39]. Previous studies have shown that SILCS-MC can improve the enrichment of active compounds relative to conventional docking approaches and has been successfully applied in prospective campaigns that identified experimentally validated compounds against multiple therapeutic targets [40–43]. Integrating a computationally expensive SILCS-MC with MolPAL may therefore make membrane-aware screening more efficient.

Overall, this comparative analysis highlights the strong influence of docking-score landscapes on the effectiveness of AL virtual screening. Rather than ranking docking programs solely by speed or recovery rate, our study emphasizes the importance of compatibility between docking engines and machine-learning surrogate models. These findings suggest a practical strategy for balancing computational efficiency and physical realism, thereby expanding the applicability of AL virtual screening to more complex biological targets, including challenging transmembrane binding sites.

## Methods

### Enamine dataset

To validate our workflow, we first tested the Enamine50k dataset published in the MolPAL github library and compared the performance with their published result [10]. The Enamine50k dataset includes a column with the SMILES string and another column with the docking score.

### ChemBridge dataset

To evaluate the performance of the active-learning (AL) workflows, we randomly sampled 50,000 compounds, represented by their SMILES strings, from the ChemBridge CORE Library Stock containing 878,427 compounds. For AutoDock Vina and SILCS-MC, the compounds were protonated using Scrubber 0.1.1 [44] and saved in SDF format. For Glide SP, the compounds were processed using the LigPrep module in Maestro. Protonation and tautomeric states were assigned using Epik at pH 7.1 ± 0.1 with the OPLS_2005 force field.

Because the preparation workflows used by Scrubber and LigPrep can generate different protonation, tautomeric, or stereochemical states, we retained only compounds for which the resulting SMILES strings were identical between the two preparations. This filtering yielded approximately 45,000 compounds with matched protonation, tautomeric, and stereochemical states. These compounds were used to generate three datasets, denoted the Vina, Glide, and SILCS datasets according to the docking method used to calculate their scores. Thus, although the initial ligand-preparation software differed, the molecular identities and chemical states represented by the SMILES strings were identical across all three datasets.

To evaluate the AL workflows on a larger chemical library, we used the complete ChemBridge CORE Library Stock. In this benchmark, all compounds were processed uniformly using Epik and LigPrep. Protonation and tautomeric states were assigned, and possible stereoisomers were generated, producing approximately 1.30 million prepared molecular entries. A single LigPrep-generated SDF file was then used as the common input for both the Vina and Glide workflows. The SDF structures were converted to PDBQT format for AutoDock Vina and to Maestro format for Glide before docking. Therefore, the Vina and Glide benchmarks for the 1.3M dataset used the same LigPrep-generated molecular structures.

#### AutoDock-Vina docking

For AutoDock Vina, the pdbqt files were docked to a defined binding site of interest using AutoDock Vina GPU 2.1 version [33]. The binding site used for this benchmark study is the pore region of the PIEZO2 protein (containing repeat A, Cap, pore, and C-terminal domain) obtained from our previous MD simulation study [32]. The binding site was prepared using MGLTools 1.5.6 and has a box size of 18 x 18 x 18 Å. Because the pore of the PIEZO2 is walled by lipids, POPC lipids near the pore are also included as part of the receptor. The Vina docking scores were saved as the ground truth for the current benchmark study.

The ALK2 and HCN1 systems were prepared in the same manner as in PIEZO2 system. For ALK2, the binding site was defined as the solvent-exposed ATP-binding site within the kinase domain, representing the same binding pocket occupied by RK-71807 in the ALK2 structure (PDB ID: 6JUX) [36], with a box size of 20 x 20 x 20 Å. For HCN1, the binding site was defined at the interface between the lipid bilayer and the cytoplasmic side, representing the same pocket occupied by ivabradine in the HCN1 structure (PDB ID: 8Y60) [37], with a box size of 22 x 22 x 22 Å.

#### Glide docking

For Schrödinger’s AL-Glide workflow, PIEZO2, ALK2, and HCN1 proteins were prepared using the Protein Preparation Workflow. The Maestro format of the 45k ligands that were prepared as mentioned above was used for docking. The binding site was defined in the same manner as the AutoDock-Vina GPU. In all cases, the proteins were held rigid while the Glide SP scoring function was used to attach all ligands to the binding site using the OPLS_2005 force field. Each generated state (including distinct stereoisomers, tautomers and protonation states) was treated as an independent ligand and docked separately using Glide SP. A maximum of 5 optimal poses were returned. The top docking score for each molecule was saved as the "ground truth" for the current benchmark study.

#### SILCS-MC docking

SILCS simulations were performed using the SILCS Small Molecule Suite (SilcsBio LLC) that uses GROMACS 2024 [45–47] for the molecular dynamics (MD) simulations and a GPU optimized oscillating chemical potential grand canonical Monte Carlo (GCMC) method for solute and water sampling [48]. The PIEZO2 open pore model [32] was embedded in a bilayer of 9:1 POPC/cholesterol along with the surrounding aqueous environment. Solutes representative of different classes of functional groups including benzene, propane, methanol, formamide, imidazole, dimethyl ether, methylammonium, and acetate were included in the simulation system. During the combined GCMC/MD simulations, these solutes and water molecules sample the full 3D space of the simulation system including the protein and membrane. The FragMaps are functional group affinity patterns based on the three-dimensional (3D) probability distributions of selected atoms in the solutes representing the different functional types as previously discussed [25]. The SILCS simulations consisted of 10 replicas of 100-ns trajectories for a total of 1 microsecond of sampling.

To predict the ligand binding poses based on FragMaps, GPU-accelerated SILCS-MC docking [35] was used. Each simulation was run with a long-local MC protocol of 1250 cycles of 10,000 Metropolis MC steps and 40,000 steps of simulated annealing (SA). The Metropolis acceptance criterion is based on the SILCS ligand grid free energy (LGFE) scores plus the CGenFF intramolecular energy. MC steps sample a wide range of binding poses with a maximum step size of 1 Å for translations, 180^◦^ for maximal rigid rotations per step and 180 for maximal dihedral angle rotations per step. This was done to ensure there is sufficient sampling of ligand poses at the binding pocket. The subsequent SA steps are designed to find a local minimum and involve a maximum step size of 0.2 Å for translations, 9$^\circ$ for maximal rigid rotations, and 9$^\circ$ for dihedral angle rotations, with gradual cooling from 300 to 0 K. The SILCS-MC runs were repeated for each molecule, with each cycle initiated using a different random seed and repeated until it completed 250 cycles or reached convergence. The lowest and average LGFE scores were obtained from the minimum scores and used for ligand scoring. The binding site was defined by a sphere centered on the PIEZO2 pore with a radius of 18.0 Å.

#### Train and test set

The SMILES strings with a score of 10,000 were removed from the dataset as they represent failed docking. All the docking datasets containing 45k SMILES strings and their respective docking scores were split into a 40k training set and a 5k test set. The docking datasets containing 1.30 M SMILES strings and their respective docking scores were split into a 1.29M training set and a 10 k test set.

### Active learning workflow

The MolPAL and Schrödinger’s workflows can be broken down into five major steps: initial batch generation, docking, training, prediction, and acquisition (Fig. 1). The initial docking batch (i.e., the original pool in Fig. 4) was created by randomly sampling molecules from the training set. The SMILES strings of the molecules and their respective docking scores are fed into the model for training, and the model then predicts the docking scores for the entire dataset. Then, greedy acquisition was employed to select the top-*k* molecules from the model prediction as the next batch for docking. This process was repeated for up to six iterations. Accumulated training data was used for each iteration. The overall AL workflow is illustrated in Fig. 1. The default hyperparameters were used for each AL model and are available at https://github.com/LynaLuo-Lab/Active-Learning-Virtual-Screening.

### Latent embedding and gradient analysis

For every surrogate model, dataset, and replicate, we extracted the 300 dimensional molecule embedding and the final layer gradient for all 40,000 molecules from the final iteration of active learning at a 1% batch size. These embeddings and gradients constituted the feature representations for the separability and clustering analyses reported in Fig. 5.

For the separability analysis, we trained a soft margin linear kernel support vector machine (SVM) [49] under fivefold stratified cross validation. Within each fold, every feature was standardized to zero mean and unit variance using statistics computed only on the training partition of that fold. Low scoring and high scoring molecules were defined as the top 25% and bottom 25% of molecules by docking score, respectively, and the middle 50% was discarded. Classification performance was quantified as the area under the ROC curve (AUC), averaged across the five folds, for both the embedding and the gradient representations.

For the clustering analysis, we reduced the Z-score normalized embeddings from 300 dimensions to a single direction by applying ridge regression against the docking score. We paired this one dimensional projection with the ground truth docking scores and re-normalized the resulting two dimensional feature matrix. We then fit a Gaussian mixture model with two components across all labeled molecules.

For the visualization, we projected the embeddings and gradients into two dimensions using Uniform Manifold Approximation and Projection (UMAP) [50]. We projected the two representations independently and L2 normalized every feature vector prior to projection. We set the number of neighbors to 30 and the minimum distance to 0.1, and we fixed the random seed across all docking engines and replicates. Each molecule was colored by its docking score after Z-score normalization within its own engine and replicate. The complete set of projections is provided in Figs. S7, S8, and S9.

### Evaluation metrics

#### Recovery rate of top-*k* scoring SMILES strings

After each iteration, the model’s recovery performance was evaluated by calculating the percentage of SMILES strings that matched those of the top-*k* scoring molecules from the ground truth (i.e., docking score), including all molecules tied at the lowest top-*k* score.

#### Coefficient of determination* R*^2^ and mean squared error (MSE)

The coefficient of determination is used to show how well our predicted docking score and the actual docking score are correlated. The Mean Squared Error measures the average square difference between the predicted values and the true values.

#### 1-D Wasserstein distance

Z-score normalization was applied to the datasets, using the respective mean and standard deviation of each dataset. Then, the 1-D Wasserstein distance was computed between the original pool’s docking score distribution and of each accumulated batch’s docking score distribution to quantitatively assess the enrichment of high-scoring molecules in our accumulated batches. The distance was computed using the *scipy* 1-D Wasserstein Distance package.

#### Chemical diversity

After each iteration, the SMILES strings of the molecules in the accumulated batches were collected into a CSV file. Using these SMILES strings, the corresponding Morgan fingerprints were generated for each molecule. Pairwise molecular similarity was then computed using the Tanimoto coefficient, yielding values between 0 and 1. The similarity values were converted into a distance matrix, which was subsequently used as input for clustering via the Butina algorithm with a distance cutoff of 0.8 [51].

## Supplementary Information

Below is the link to the electronic supplementary material.

Supplementary file 1 (pdf 17042 KB)

## Data Availability

The Enamine50k benchmark dataset can be downloaded from https://github.com/coleygroup/molpal/tree/main/data. The Chembridge45k and Chembridge1.3M datasets with docking scores from Vina, Glide, and SILCS-MC docking are available at https://github.com/LynaLuo-Lab/Active-Learning-Virtual-Screening.
