# Supplementary material for: Docking-score landscapes shape active-learning performance across Vina, Glide, and SILCS
Source: J Comput Aided Mol Des. 2026 Sep 11;40(1):231. doi: 10.1007/s10822-026-00927-x (PMC13569507; doi:10.1007/s10822-026-00927-x)
Supplement: Supplementary file 1 [file 10822_2026_927_MOESM1_ESM.pdf]

Supporting Information

Joseph Chung,[†] Aashish Bhatt,[†] Jacob Ede Levine,[‡] Yichun Lin,[†] Mingtian Zhao,[¶] Alexander D. MacKerell, Jr.,[*,¶] Sunhwan Jo,[*,†] Sai Chandra Kosaraju,[*,‡] and Yun Lyna Luo[*,†]

†*Department of Biotechnology and Pharmaceutical Science, Western University of Health Sciences, Pomona, CA, USA*

‡*Department of Computer Science, California State Polytechnic University, Pomona, CA, USA*

¶*Department of Pharmaceutical Science, University of Maryland, School of Pharmacy, Baltimore, MD, USA*

E-mail: alex@outerbanks.umaryland.edu; sunhwanj@gmail.com; skosaraju@cpp.edu; luoy@westernu.edu

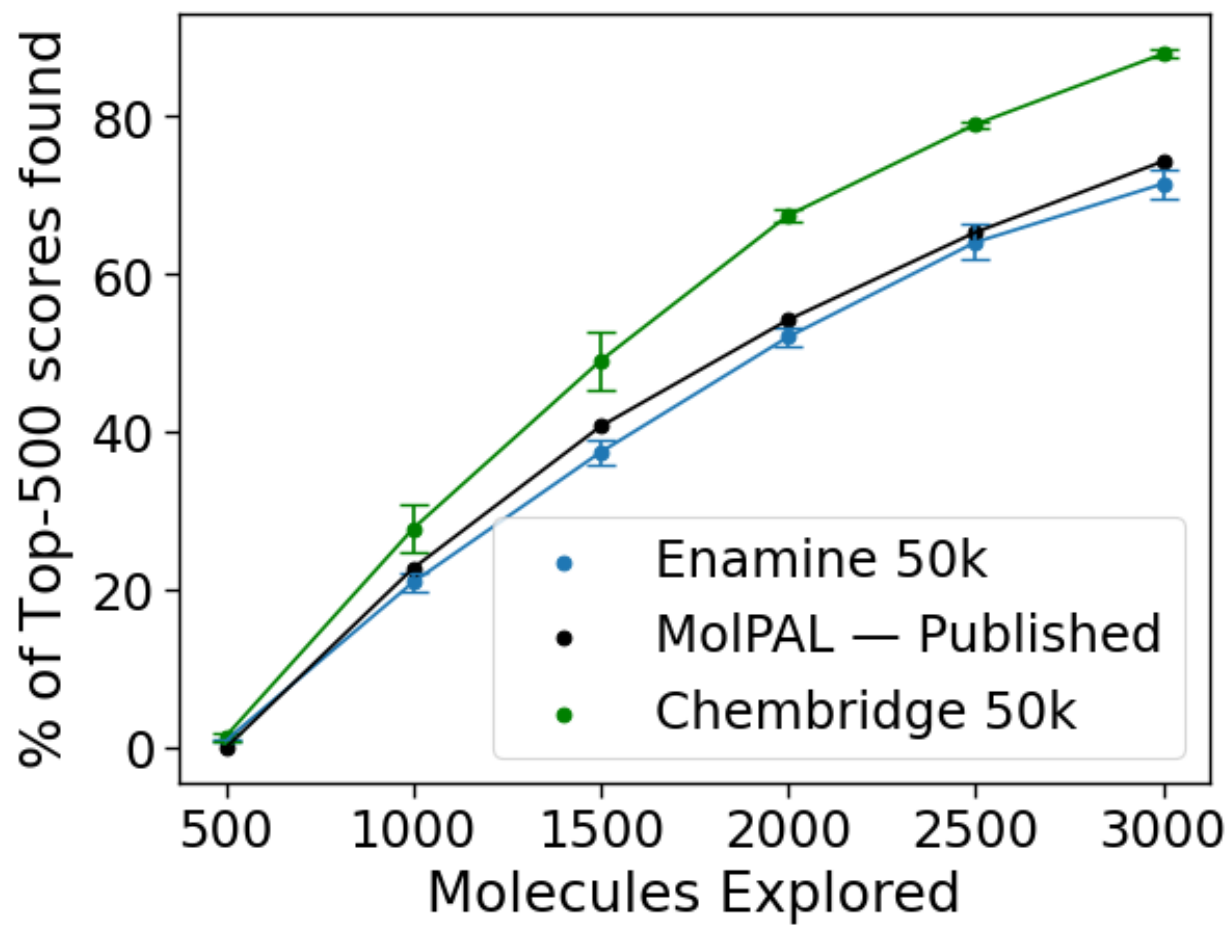

Figure S1.Percent of Top-500 scores collected from either the Enamine50k or Chembridge 50k dataset with a 1% Batch Size (500 molecules) Exploration. Enamine 50k and published MolPAL results used Enamine50k dataset.

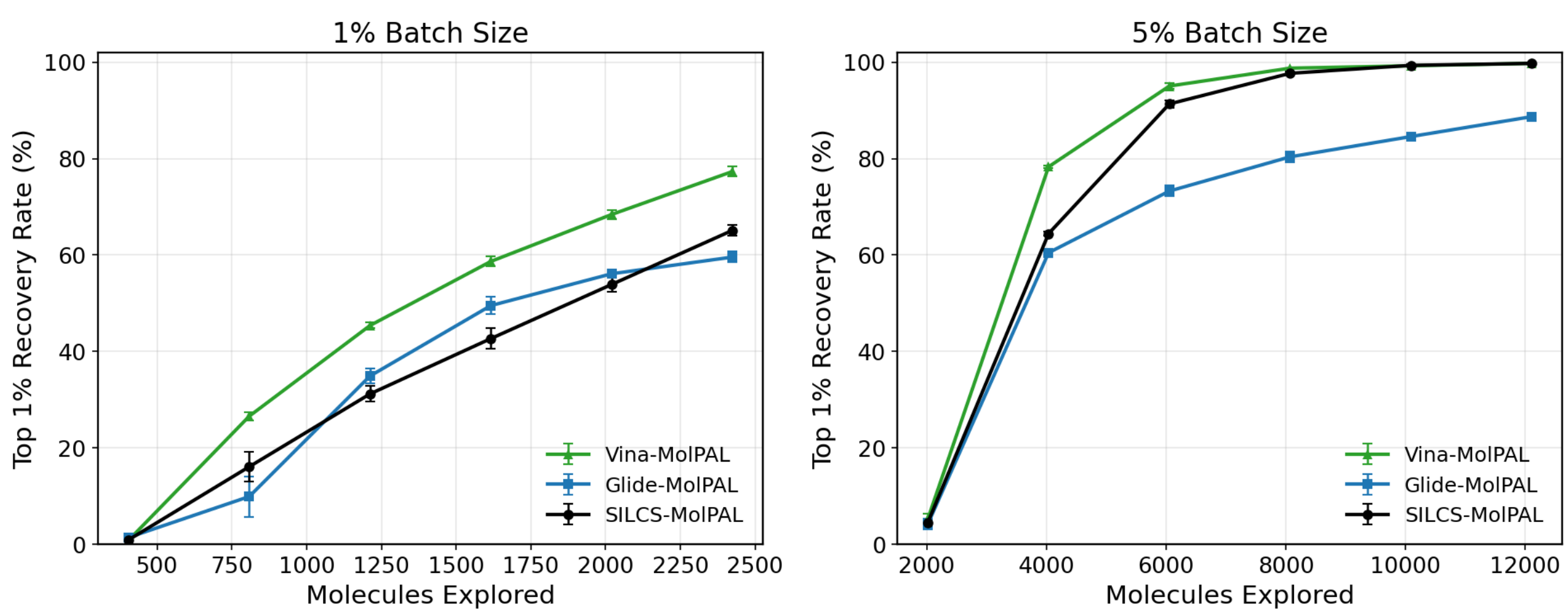

Figure S2. Percent of the top-1% scoring SMILES string recovered as a function of number of molecules explored with either a 1% batch size (400 molecules) or a 5% batch size (2000 molecules) exploration for the 40k dataset using Chemprop v2.

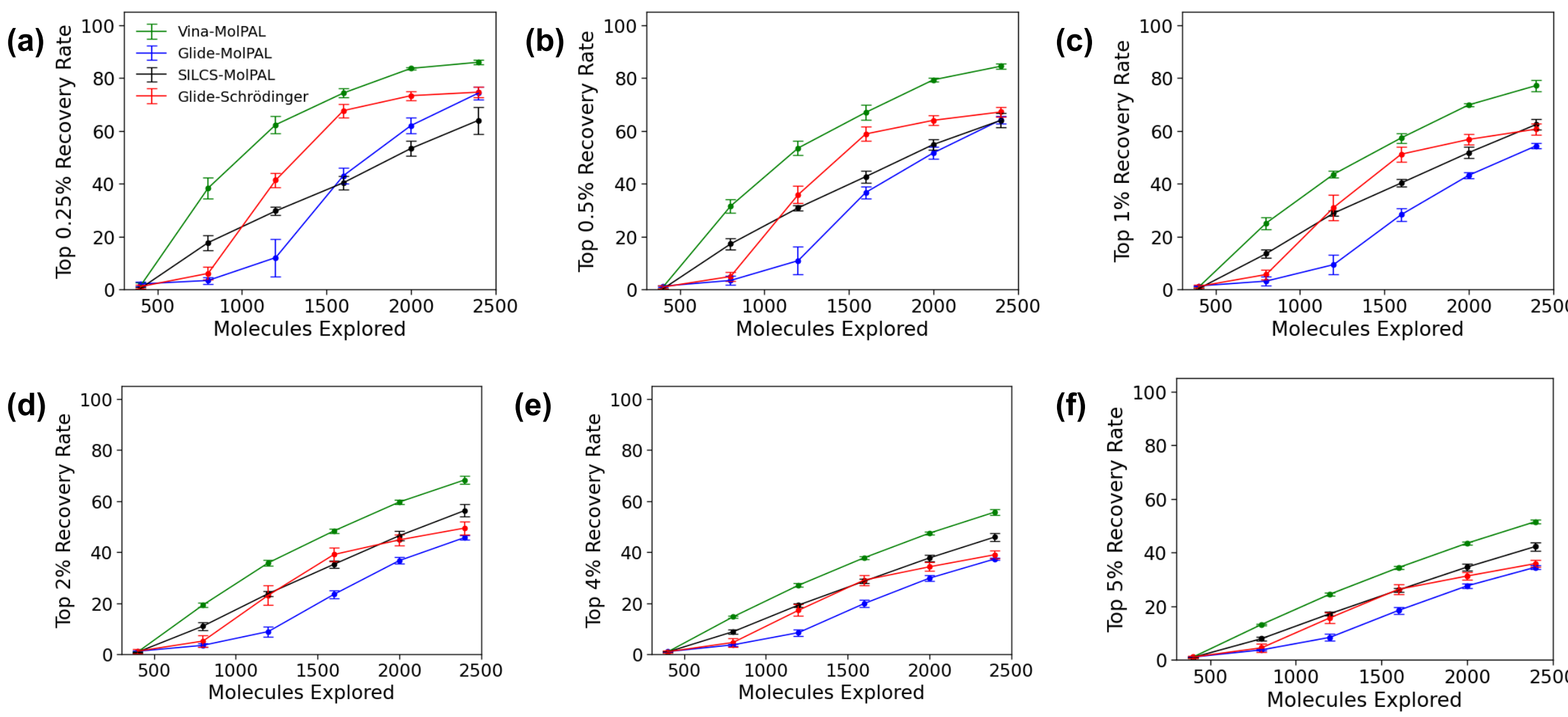

Figure S3. Percent of the top-k percent scoring SMILES string found as a function of number of molecules explored over 3 replicas with a 1% batch size exploration. (a) top-0.25% (b) top-0.50% (c) top-1% (d) top-2% (e) top-4% (f) top-5%

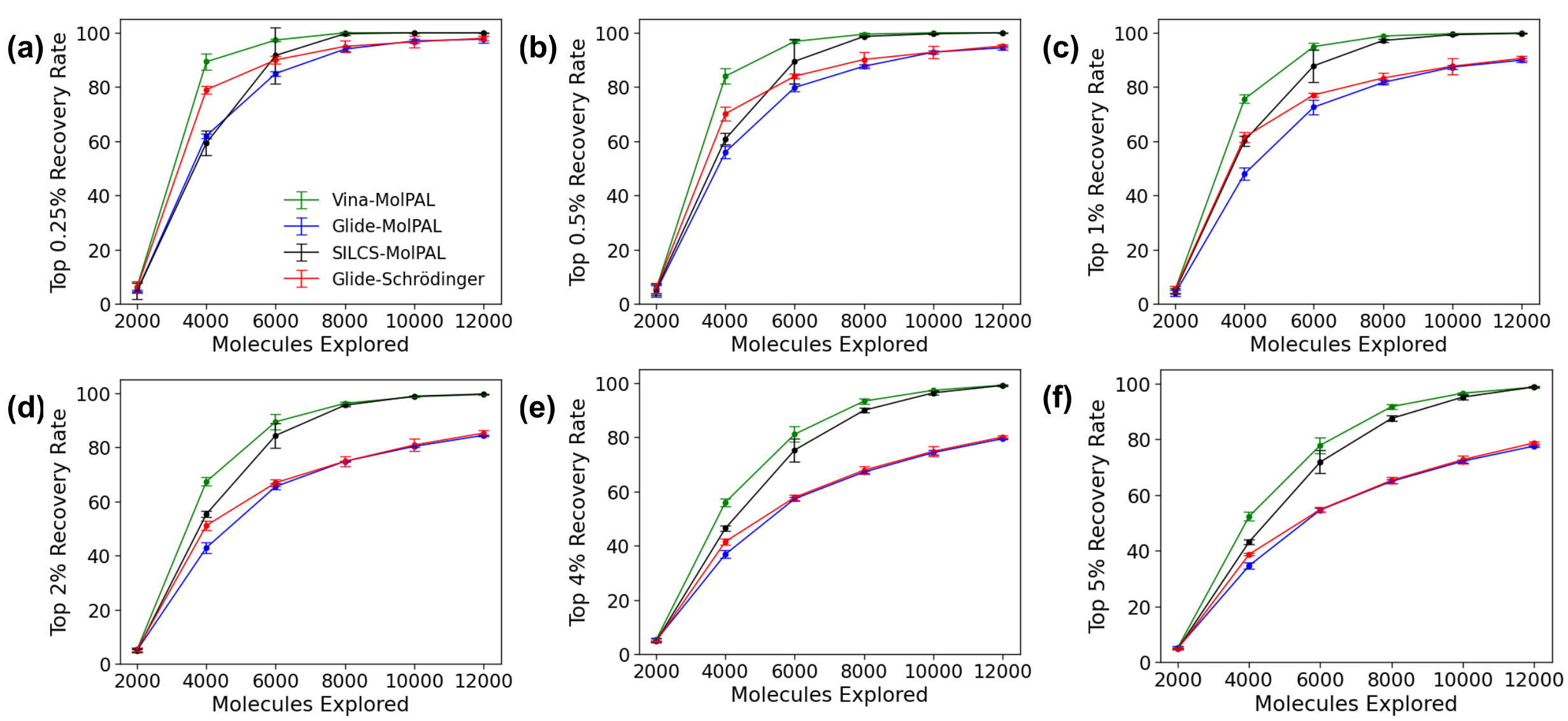

Figure S4. Percent of the top-k percent scoring SMILES strings found as a function of number of molecules explored over 3 replicas with a 5% batch size exploration (a) top-0.25% (b) top-0.50% (c) top-1% (d) top-2% (e) top-4% (f) top-5%

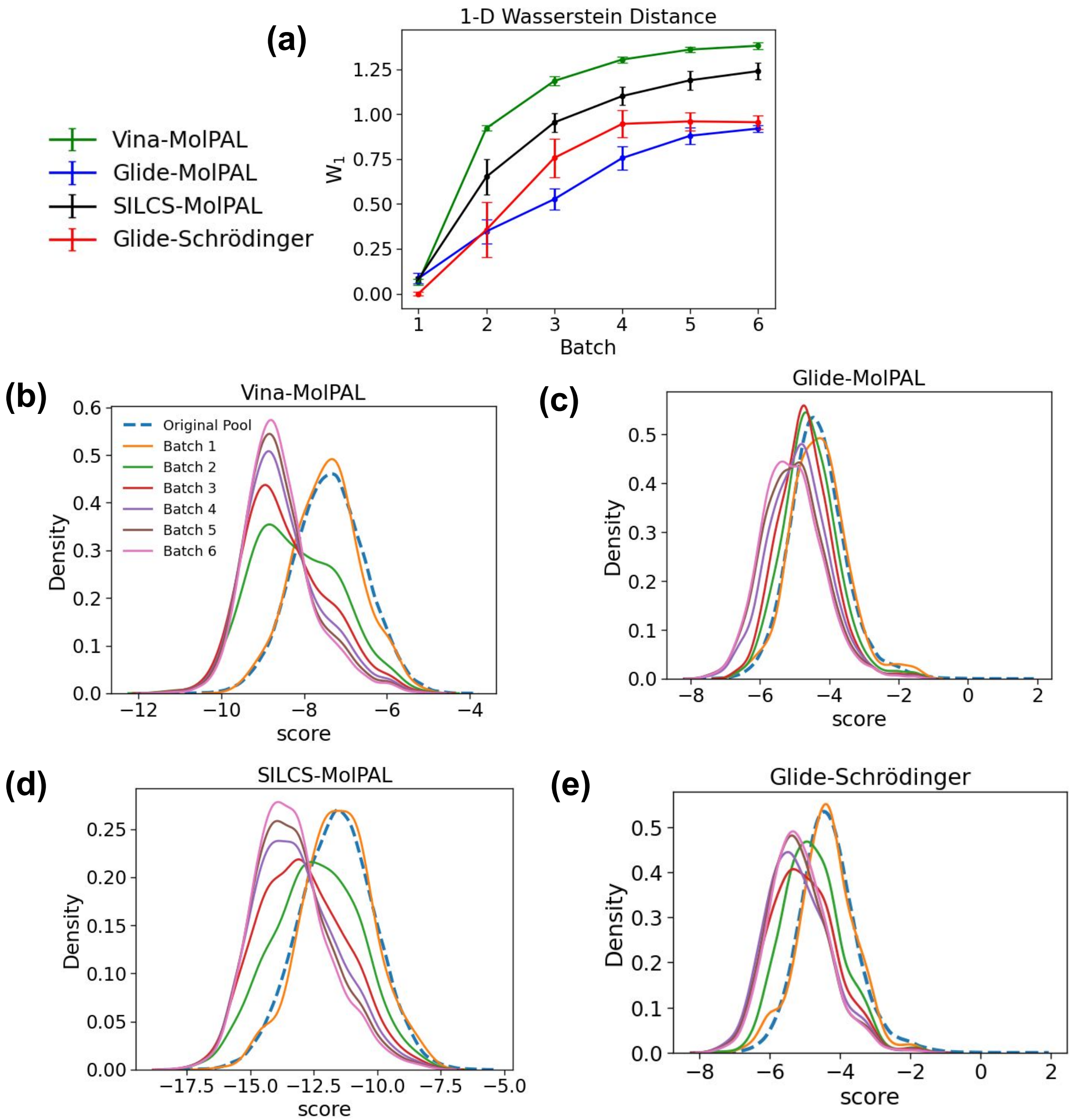

Figure S5. 1% Docking Score Distributions (a) 1-D Wasserstein distance was calculated from the original pool to the distribution of each of the accumulated batches starting from batch 2 across three replicas using a 1% batch size. (b–e) Dotted lines represent the distributions of the full datasets, while bold lines indicate the distributions of the accumulated training sets across batches for Replica 1 using a 1% batch size. The panels correspond to Vina-MolPAL, Glide-MolPAL, SILCS-MolPAL, and active-learning Glide, respectively.

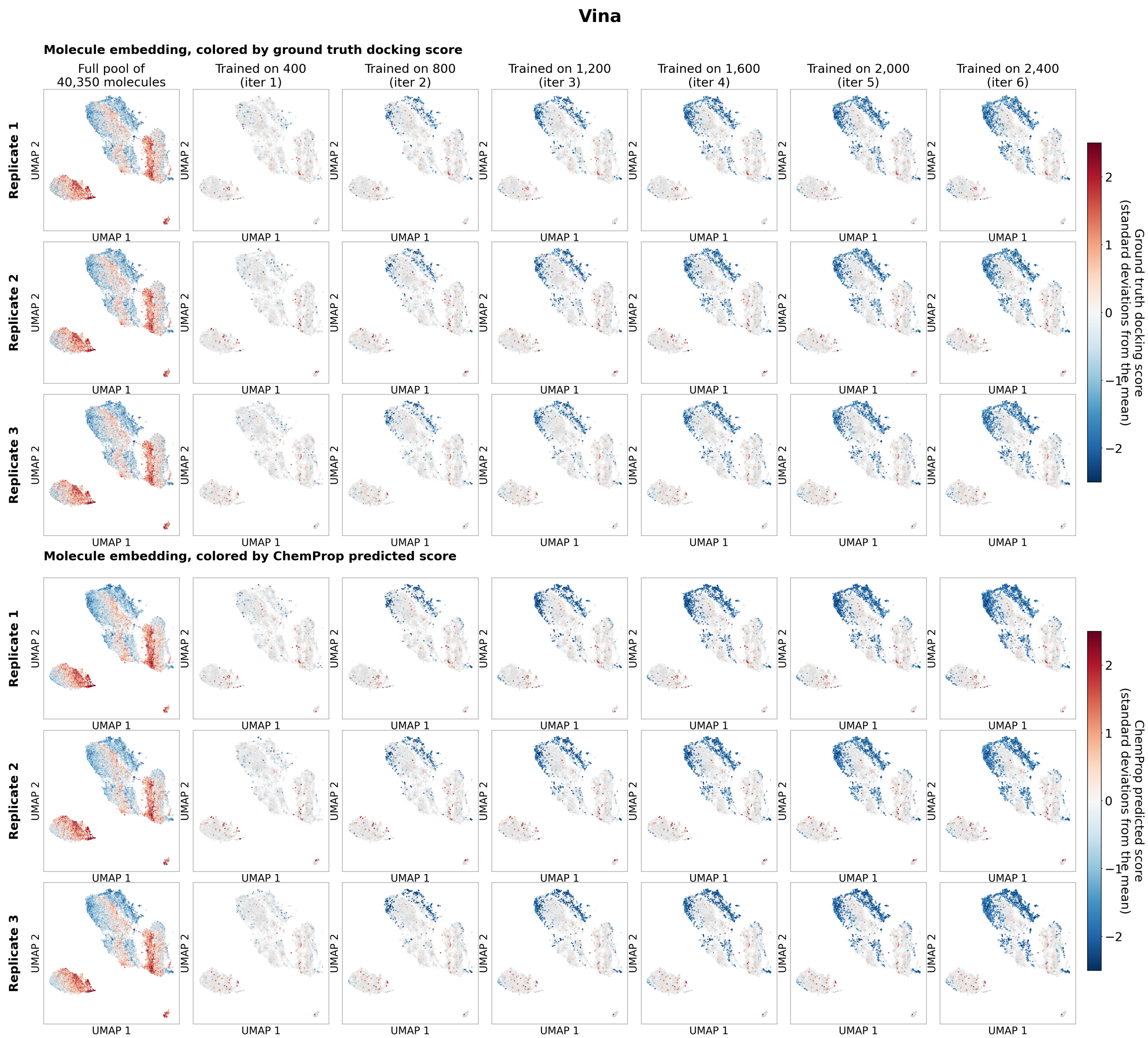

Figure S6. UMAP Visualization of Chemprop's molecular embeddings for Vina-MolPAL (see Methods on dimension reductionality details). The underlying gray scatter points represent the full 40k Vina dataset projected into the latent embedding space. Colored points overlaying the dataset represent molecules sampled during the active learning iterations, colored by either their true normalized docking score (top) or the corresponding Chemprop-predicted score (bottom).

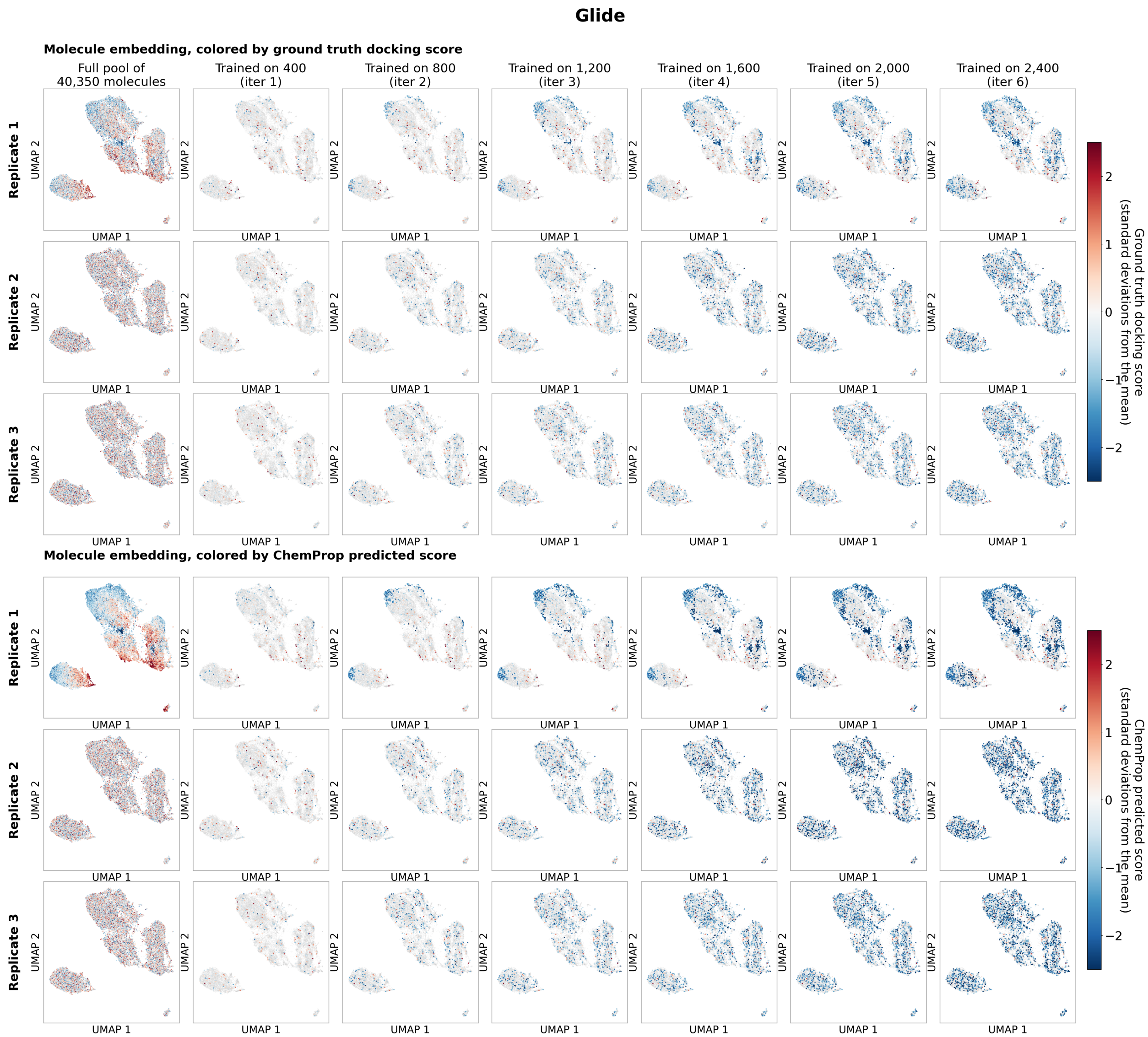

Figure S7. UMAP of Chemprop's Molecular Embeddings for the Glide-MolPAL. See figure details in Figure S7.

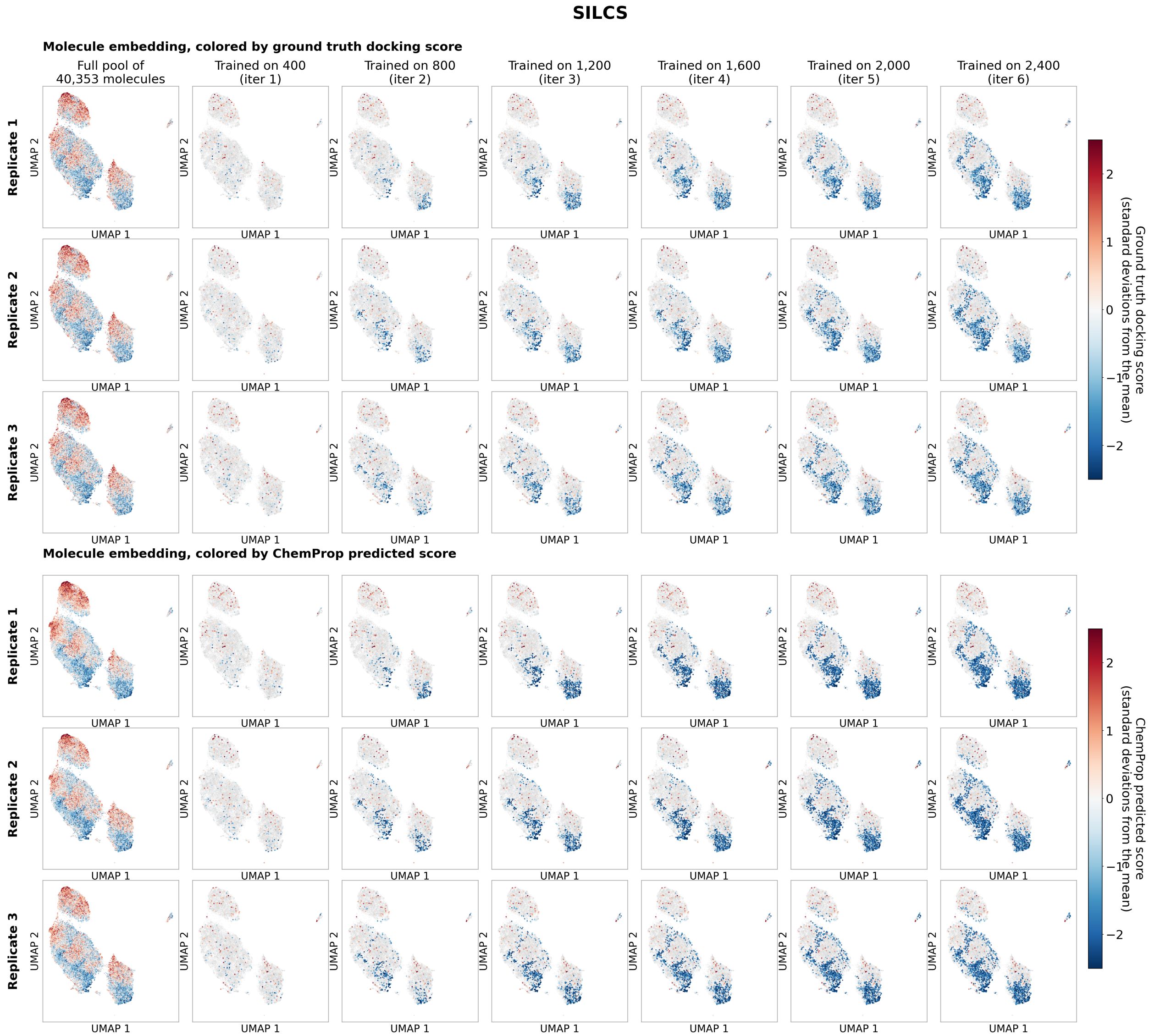

Figure S8. UMAP of Chemprop's Molecular Embeddings for the SILCS-MolPAL. See figure details in Figure S7.

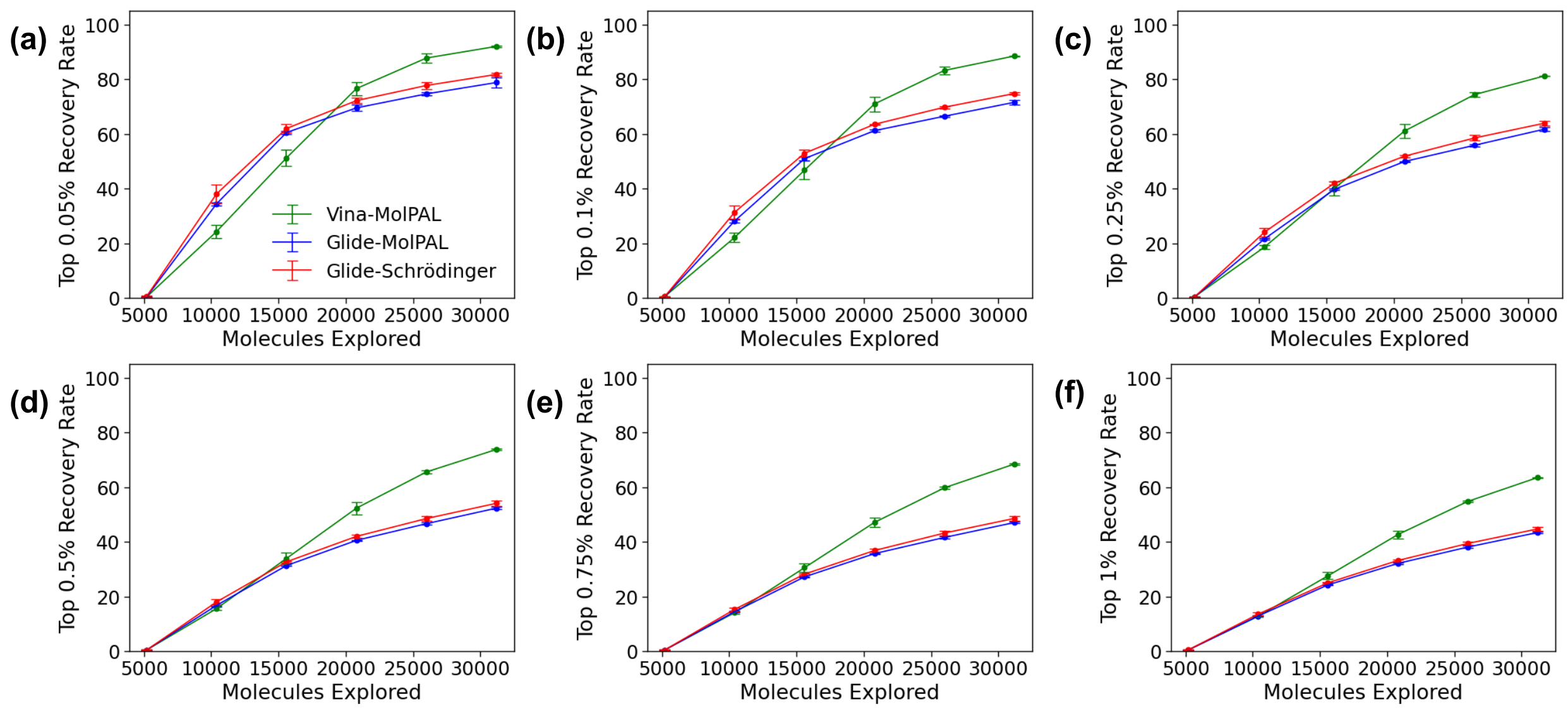

Figure S9. Percent of the top-k percent scoring SMILES string found as a function of number of molecules explored over 3 replicas with a 0.4% batch size exploration for a 1.3M dataset (a) top-0.05%(b) top-0.1% (c) top-0.25% (d) top-0.5% (e) top-0.75% (f) top-1%

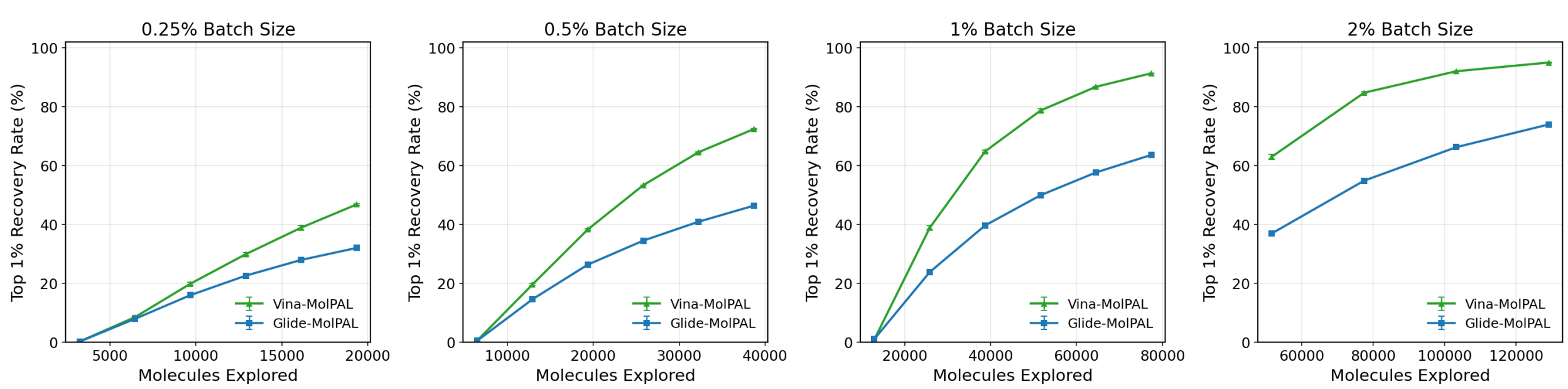

Figure S10. Percent of the top-1% percent SMILES string recovered as a function of number of molecules explored over 3 replicas with varying batch size exploration for a 1.3M dataset using Chemprop v2.

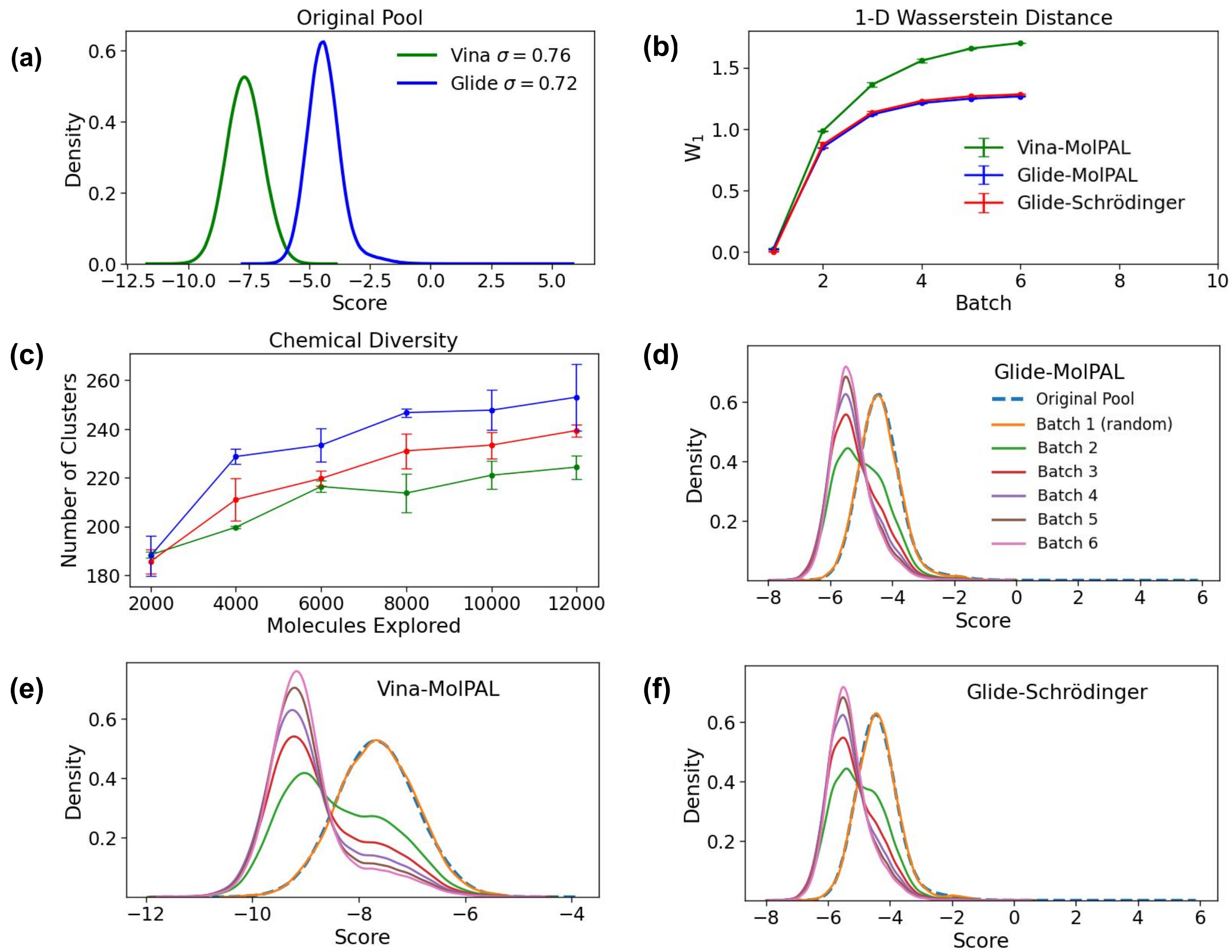

Figure S11. Distribution and Diversity for 1.3M Dataset (a) Docking score distributions for 1.3M dataset (i.e. original pool) over Vina and Glide (b) 1-D Wasserstein distance (W_1) was calculated from the original pool to the distribution of each of the accumulated batches across three replicas using a 0.4% batch size. (c) Diversity of molecules in accumulated batches as function of molecules explored using a 0.4% batch size (5.2k molecules). Molecules were clustered using Butina Clustering using their Morgan fingerprints with a distance cutoff of 0.8 (d-f) Dotted lines represent the distributions of the original pool, while bold lines indicate the distributions of the accumulated training sets across batches from replica 1 using a 0.4% batch size. The panels correspond to Glide-MolPAL, Vina-MolPAL, and active learning Glide, respectively.

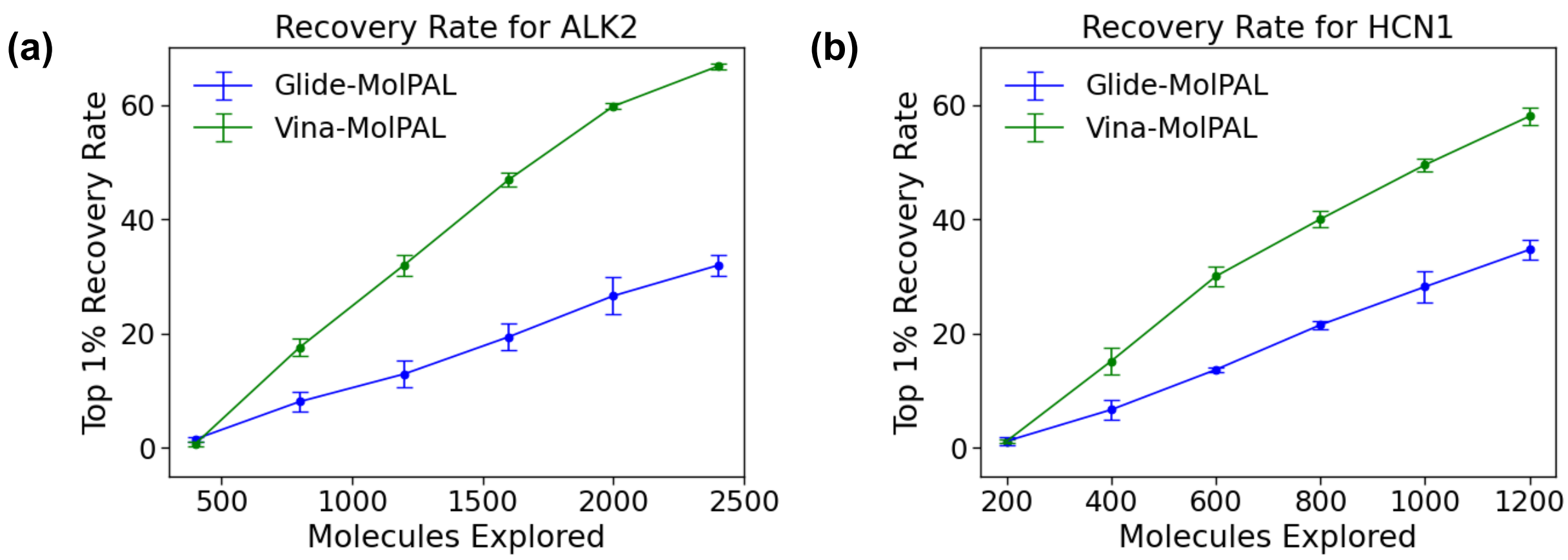

Figure S12. Percent of the top-1% scoring SMILES string recovered as a function of molecules explored with a 1% batch size across distinct protein targets. a) Kinase ALK2. 1% Recovery Rate using a randomly sampled 40k dataset from 1.3M Chembridge Library. Batch size is 400 molecules b) Voltage-gated ion channel HCN1. 1% Recovery Rate using a randomly sampled 20k dataset from 1.3M Chembridge Library. Batch size is 200 molecules.

Glide 40k — Recovery@1%

Method	Mean	±Std	Rep 1	Rep 2	Rep 3
**1% Batch (404 mols × 6 rounds)**					
ChemProp — Greedy MSE	59.57	1.11	60.89	59.65	58.17
ChemProp — Greedy MVE	59.08	1.65	58.17	57.67	61.39
ChemProp — UCB β=2 MVE	59.32	1.11	60.89	58.42	58.66
**5% Batch (2018 mols × 6 rounds)**					
ChemProp — Greedy MSE	88.70	0.31	88.61	88.37	89.11
ChemProp — Greedy MVE	88.12	0.35	87.62	88.37	88.37
ChemProp — UCB β=2 MVE	89.85	0.81	89.85	90.84	88.86

Table S1. Comparison of Recovery Rate of top-1% scoring SMILES strings using different acquisition metrics for Glide-MolPAL over three replicas. Greedy acquisition was tested using either the Mean-Squared Error (MSE) or Mean-Variance Estimation (MVE) uncertainty metric. Upper Confidence Bound (UCB) acquisition was tested with a β=2 and MVE uncertainity metric.
